# A Causal Inference Approach to Mediation Analysis in Vitreomacular Traction: How Much Does Traction Resolution Mediate Functional Outcomes?

**DOI:** 10.3390/jmahp12040022

**Published:** 2024-09-30

**Authors:** Benedicte Lescrauwaet, Stijn Vansteelandt, Timothy L. Jackson, SriniVas R. Sadda, Luc Duchateau

**Affiliations:** 1 Biometrics Research Group, Ghent University, 9000 Ghent, Belgium; luc.duchateau@ugent.be; 2 Department of Applied Mathematics, Computer Science and Statistics, Ghent University, 9000 Ghent, Belgium; stijn.vansteelandt@ugent.be; 3 Faculty of Life Science and Medicine, King’s College London, London SE5 9RS, UK; t.jackson1@nhs.net; 4 Doheny Image Reading Center, Doheny Eye Institute, Los Angeles, CA 91103, USA; ssadda@doheny.org

**Keywords:** anatomic outcomes, functional outcomes, biomarker, causal mediation analysis, patient-reported outcomes, vitreomacular adhesion/traction

## Abstract

Modern mediation analysis techniques supplement the primary intention-to-treat analysis with the aim to shed light onto the treatment mechanism. We investigate to what extent the anatomic marker vitreomacular adhesion resolution (VMAR) mediates vision benefits, comparing ocriplasmin vs. a sham regimen. A causal mediation analysis is applied to randomized trial data including 218 participants with vitreomacular traction. Logistic regression models are used to estimate the total treatment effect (TTE) on binary outcomes. Outcomes, assessed at month 24, included visual acuity improvement (VA-I): ≥2-line increase in VA; visual function questionnaire improvement (VFQ-I): ≥5-point increase in the 25-item visual function questionnaire composite score (VFQ-25cs); visual function improvement (VF-I): defined as either a VA-I or a clinically meaningful improvement in the VFQ-25cs. Quantity of interest is the breakdown of TTE into an indirect (through VMAR) and direct effect to estimate the extent to which the TTE is transmitted through the mediating variable (VMAR) vs. other pathways. Causal effects are expressed as risk differences. Indirect effects for VFQ-I, VA-I, and VF-I are 5.7%, 11.8%, and 5.2%, respectively, representing the increase in the probability of a vision improvement if VMAR status were changed for each participant to the extent that it is affected by ocriplasmin. The direct effects are 8.3%, 12.1%, and 24.1% respectively, capturing the effect of treatment on the probability of a vision improvement if ocriplasmin left each participant’s VMAR status unchanged. The relative treatment effect of ocriplasmin on the functional outcome VA-I is to a large extent the result of its effect on VMAR, while an improvement in the patient-reported outcomes VFQ-I or VF-I was only partially mediated by VMAR.

## 1. Introduction

Most clinical trials of new eye treatments rely on visual acuity (VA) eye charts to measure treatment success. However, for some ocular conditions, eye charts fail to fully capture patient symptoms [1]. This is particularly true for vitreomacular traction (VMT), a disorder of the vitreoretinal interface in which abnormal vitreous traction pulls on the macula resulting in dysfunction and damage to the photoreceptors and connecting neurons important for central vision [2,3]. While a VA chart is the most commonly used tool to assess visual function in clinical practice, it does not capture the entirety of visual disturbances in VMT, such as the impact of metamorphopsia (distorted vision) and micropsia on everyday activities, with attendant effects on quality of life. Anatomic outcomes are therefore often used to measure treatment success. The premise is that visual symptoms improve as the anatomic integrity of the vitreoretinal interface is restored [1]. Regulatory authorities previously appraised vitreomacular adhesion resolution (VMAR), an anatomic endpoint, as a surrogate marker for the prevention of vision deterioration despite limited experience of its reliability to substitute for a clinically meaningful endpoint [4,5]. Yet, Health Technology Assessment (HTA) agencies often take a more conservative approach to the use of surrogate endpoints to support their reimbursement recommendations with a preference for patient-relevant outcomes and limiting the use of surrogate outcomes to validated measures [6]. Nevertheless, treatment resulting in VMAR has been shown to correlate well with an improvement in VA and avoidance of surgery, supporting the clinical relevance of this anatomic endpoint [4,7,8]. In addition to assessing visual function, the National Eye Institute visual function questionnaire-25 (VFQ-25), a patient-reported outcome (PRO) measure, is often administered to evaluate changes in vision-related function in VMT [9,10,11].

Randomized controlled trials (RCTs) are the gold standard for estimating causal treatment effects and focus on the questions of whether and how much a treatment impacts on outcomes [12]. Causal mediation analysis, on the other hand, is a statistical framework to gain insight into why and how a treatment works [13]. A causal mechanism is the process through which one variable, exposure, causally affects another variable, outcome*,* through one or more intermediate variables also termed mediators. The inferential goal is to break down the total treatment effect into an indirect (mediated) effect, the hypothesized causal mechanism, and a direct (unmediated) effect, representing all the other—often unknown—mechanisms [14]. Compared with RCTs, where treatment assignment is randomized, mediation analysis requires strong confounding assumptions for the estimates of direct and indirect effects to be interpretable causally [15]. Indeed, the randomization of treatment alone does not suffice to identify causal mediation effects. This is because mediation analysis is about the exposure changing the mediator and that change in the mediator affecting the outcome. Thus, to estimate the indirect effect, we must know how much the mediator affects the outcome, where the mediator is an observed, not randomly assigned post-treatment covariate. The control of confounding of covariates that affect both the mediator and outcome is required to interpret mediation effects as causal: more specifically, we will assume that among participants who have the same treatment and comparable characteristics, the mediator can be regarded as if it were randomly assigned [13,16].

This article is motivated by a secondary analysis of the Ocriplasmin for Treatment for Symptomatic Vitreomacular Adhesion Including Macular Hole (OASIS) clinical trial, which was originally set up to assess the primary intent-to-treat effect of intravitreal injection of ocriplasmin on VMAR in VMT [17]. Treatment with ocriplasmin vs. control was associated with a higher rate of VMAR [8,17]. Also, success on this anatomic endpoint was associated with improved functional outcomes and PROs, more specifically vision-related functioning, and visual function response [9,18,19]. A mediation analysis can explain to what degree the treatment effect of ocriplasmin vs. control on vision outcomes is due to VMAR vs. other pathways. We use the OASIS trial as a case study to explain how the causal inference approach to mediation analysis works in the setting of a population treated for VMT, and to inform scientists how this quantitative research can be relevant in other conditions [15]. We apply modern mediation analysis techniques to investigate the interrelationships between interventions (ocriplasmin vs. control) and vision outcomes to elucidate the role of VMAR and estimate the magnitude of the mediated and direct effects. Presuming the surrogate marker VMAR is an endpoint for treatment success in VMT, we aim to understand how this anatomic surrogate is associated with an improvement in patient-relevant functional outcomes and PROs.

## 2. Materials and Methods

We conducted a secondary analysis using data from participants of the OASIS trial. The OASIS pivotal trial (clinicaltrials.gov identifier: NCT01429441) was a phase 3b, randomized, sham-controlled, double-masked, multicenter clinical trial designed to provide longer-term outcomes on the efficacy and safety profile of ocriplasmin versus sham. Complete trial methods and study results have been previously described [17]. A sample of 220 participants were recruited from 25 different ophthalmic settings in the United States between November 2011 and October 2014. All participants had a clinical diagnosis of VMT defined as the presence of vitreomacular adhesion (VMA) that was related to decreased visual function. Eyes had either VMT alone, or VMT associated with a full-thickness macular hole (FTMH). Participants received a single intravitreal injection of 125 micrograms of ocriplasmin or a sham injection and were assessed at multiple timepoints up to month 24. The primary efficacy endpoint was the proportion of participants with VMAR at day 28. If the underlying condition had not resolved rescue therapy, vitrectomy was provided. The study adhered to the provisions of the guidelines of the World Medical Association Declaration of Helsinki. Participants provided written informed consent before enrollment in the trial [17]. Participant characteristics, efficacy outcomes and completion rate are reported in supplement S1: OASIS study characteristics (Table S1).

The present mediation analysis follows the ‘A Guideline for Reporting Mediation Analyses of Randomized Trials and Observational Studies (AGReMA)’ statement [20].

### 2.1. OASIS Variables for Mediation Analysis

**Figure 1:**
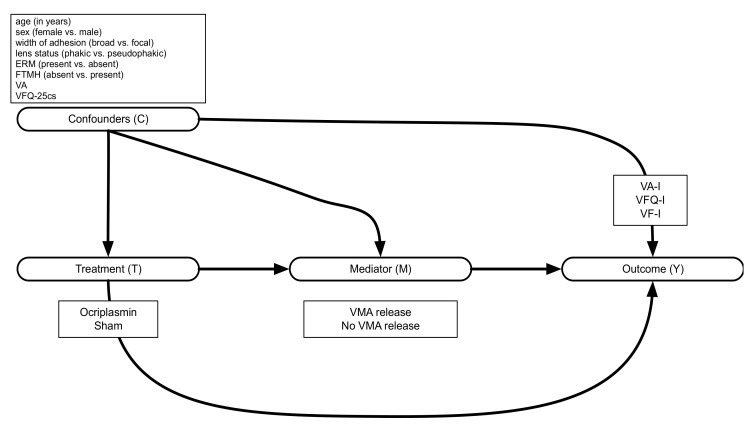
Causal diagram visualizing the relations between treatment *T*, mediator *M*, outcome *Y*, and confounders *C.* ERM: epiretinal membrane; FTMH: full-thickness macular hole; VA: visual acuity; VFQ-25cs: visual function questionnaire-25 composite score; VMA: vitreomacular adhesion; VA-I: visual acuity improvement; VFQ-I: visual function questionnaire improvement; VF-I: visual function improvement.

We will let *T* denote the exposure of interest, *Y* a dichotomous outcome, and *M* a candidate mediator. We let *C* denote a set of baseline covariates not affected by the exposure. The relationships among these variables are depicted in Figure 1.

Exposure T*:* Participants are exposed to ocriplasmin (*t* = 1) or sham (*t* = 0).

Mediator M*:* In a mediation analysis, the question is how (through which mechanism) exposure affects the outcome. A key question is the extent to which the effect of ocriplasmin on vision outcomes is transmitted through the mediating variable vs. through other pathways. The candidate mediator variable M corresponds to VMAR at day 28, a binary variable defined as success (M = 1) or failure (M = 0). This mediation analysis seeks to assess the extent to which the effect of ocriplasmin is mediated by VMAR.

Outcome Y*:* The outcome variables considered are VA improvement (VA-I), i.e., VA-response defined as a ≥2-line improvement in VA; VFQ improvement (VFQ-I), i.e., VFQ-response defined as a ≥5-point improvement in VFQ-25 composite score (VFQ-25cs); visual function improvement (VF-I), i.e., a composite endpoint defined as either a VA-I or a clinically meaningful improvement (≥3.7 points) in the VFQ-25cs [19]. Outcomes are evaluated at month 24 and are binary variables (success = 1; failure = 0). If participants require a vitrectomy prior to month 24, any of the three vision outcomes is considered an outcome failure.

Baseline covariates (confounders) *C:* To estimate direct and indirect effects in randomized trials, it is essential to control for confounders (i.e., common causes) of the mediator–outcome association (supplement S2: Confounding assumptions for mediation analysis) [21,22]. In this article, we restrict this adjustment to the covariates age, sex, width of adhesion, lens status, epiretinal membrane (ERM), and FTMH, which are known prognostic factors that predict VMAR [23,24,25]. Measurements of the continuous outcomes VA and VFQ-25cs are also included [26]. By limiting the adjustment to baseline covariates, we thus assume that there are no post-treatment variables that are common causes of the mediator–outcome relationship [21].

### 2.2. Statistical Methods

#### 2.2.1. Defining Mediated and Direct Effects in Mediation Analysis

The counterfactual framework for mediation analysis is based on counterfactual outcomes: one outcome that would be observed if the participant receives sham (*t* = 0) and the other outcome that would be observed if that same participant receives ocriplasmin (*t* = 1). Only one of these counterfactual outcomes can be observed for a single participant *i*. The two counterfactual outcomes for participant *i* are given by $Y_{i}(0)$ and $Y_{i}(1)$ for the observation under sham and ocriplasmin, respectively, and the observed outcome is denoted by $Y_{i}(T_{i})$. The total treatment effect for participant *i* corresponds to ${\tau_{i}=Y}_{i}\left(1\right)-Y_{i}(0)$. The mediation analysis investigates how much of this total treatment effect is due to M. We denote by $M_{i}$ the mediator status for participant *i*, with $M_{i}(0)$ and $M_{i}(1)$ corresponding to the mediator status when participant *i* receives sham or ocriplasmin, respectively. Again, only one of these counterfactual mediator values is observed for a single participant *i*, denoted by $M_{i}(T_{i})$. As an example, we consider the outcome VF-I. For a treated individual, the observed (factual) VF-I is denoted by $Y_{i}(1)$, or equivalently $Y_{i}\left(1,M_{i}\left(1\right)\right)$, whereas $Y_{i}\left(1,M_{i}\left(0\right)\right)$ corresponds to the unobservable (counterfactual) VF-I that would occur if that same participant, although receiving ocriplasmin, would have a VMAR status as if sham was given, i.e., $M_{i}(0)$.

Bringing it all together, for each participant *i* there are four counterfactual outcomes, $Y_{i}(0,M_{i}\left(0\right))$, $Y_{i}(0,M_{i}\left(1\right))$, $Y_{i}(1,M_{i}\left(0\right))$, and $Y_{i}(1,M_{i}\left(1\right))$. Again, only one of these, $Y_{i}(T_{i},M_{i}\left(T_{i}\right))$, is observed for a single participant *i*.

Based on these counterfactual outcomes, we define the indirect (mediated) effects (IEs) as

$$\left\{\begin{array}{c} {\;IE}_{i}\left(1\right)=Y_{i}\left(1,M_{i}\left(1\right)\right)-Y_{i}\left(1,M_{i}\left(0\right)\right)\\ {IE}_{i}\left(0\right)=Y_{i}\left(0,M_{i}\left(1\right)\right)-Y_{i}\left(0,M_{i}\left(0\right)\right) \end{array}\right.$$

${\;IE}_{i}$ represents what would happen to VF-I if VMAR status were changed for each participant *i* to the extent that it is affected by ocriplasmin for that participant *i* (i.e., a change in VF-I if the mediator status changed from ${\;M}_{i}\left(0\right)$ to $M_{i}(1)$. ${IE}_{i}\left(1\right)$ is the difference between the value of two counterfactuals when receiving ocriplasmin and represents the effect on VF-I as a result of the change in VMAR status induced by ocriplasmin, while other possible effects of ocriplasmin are eliminated. Similarly, the ${IE}_{i}\left(0\right)$ is the difference between the value of two counterfactuals when receiving the sham-treatment. It expresses the sham effect on VF-I that is mediated through VMAR (i.e., how much the VF-I would change if participant *i* had received sham and the mediator changed from its natural level had participant *i* been assigned to sham ${\;M}_{i}\left(0\right)$, to its natural level had participant *i* been assigned to ocriplasmin $M_{i}(1)$.

Based on the counterfactual outcomes, we define the direct effects (DEs) as

$$\left\{\begin{array}{c} {DE}_{i}\left(1\right)=Y_{i}\left(1,M_{i}\left(1\right)\right)-Y_{i}\left(0,M_{i}\left(1\right)\right)\\ {DE}_{i}\left(0\right)=Y_{i}\left(1,M_{i}\left(0\right)\right)-Y_{i}\left(0,M_{i}\left(0\right)\right) \end{array}\right.$$

These capture the effect on VF-I if the intervention left each participant’s VMAR status unchanged. In the OASIS mediation analysis, ${DE}_{i}\left(1\right)$ represents the direct (unmediated) effect of ocriplasmin on participant *i* ’s VF-I. This expresses the change in VF-I that would be observed in participant *i* if his/her treatment changed from sham $Y_{i}(0,M_{i}\left(1\right))\;$to ocriplasmin $Y_{i}\left(1,M_{i}\left(1\right)\right)$, but the VMAR status is kept at the level it would naturally have taken in the presence of ocriplasmin, i.e., $M_{i}(1)$. The ${DE}_{i}\left(0\right)$ has the same interpretation but holding participant *i’*s VMAR status constant at $M_{i}(0)$, and represents the causal effect of ocriplasmin on VF-I through all other possible mechanisms than VMAR.

The total treatment effect (${TTE}_{i}$) for a single participant *i* can be broken down in terms of these (in)direct effects as follows:

$$\begin{array}{cc} {TTE}_{i} & {=Y}_{i}\left(1\right)-Y_{i}\left(0\right)\\ & {=Y}_{i}\left(1,M_{i}\left(1\right)\right)-Y_{i}\left(0,M_{i}\left(0\right)\right)\\ & =Y_{i}\left(1,M_{i}\left(1\right)\right)-Y_{i}\left(1,M_{i}\left(0\right)\right){+Y}_{i}\left(1,M_{i}\left(0\right)\right)-Y_{i}\left(0,M_{i}\left(0\right)\right)\\ & ={IE}_{i}\left(1\right){+DE}_{i}\left(0\right) \end{array}$$

An alternative way of breaking down the TTE is illustrated in supplement S3: Breakdown of total treatment effect (Figure S3) [13,21,27,28,29].

While the (in)direct effects are now defined, these cannot be calculated since we observe only one counterfactual vision outcome for a single participant *i*. However, different techniques exist to generate the four counterfactual outcomes for each participant and obtain estimates of the average (in)direct effects at the study population level.

#### 2.2.2. Estimating the Mediated and Direct Effects

The estimation of average (in)direct effects will be based on the so-called mediation formula which involves two regression models [30]. First, the mediator model predicts for each participant and for each treatment the probability of a successful response for the mediator. Based on these probabilities each participant is randomly classified as successful responder or not (on ocriplasmin or sham). Hence, for each participant, two counterfactual mediator values are generated. Second, these counterfactual mediator values are used in the fitted outcome model to predict for each participant and for each treatment the probability of a successful response for the outcome. The obtained probabilities are used in a Monte Carlo simulation to estimate the four counterfactual outcomes from which the (in)direct effect for each participant can be derived, and subsequently averaged over the study participants. The counterfactual values represent random variables; therefore, each simulation may produce different results for the counterfactuals. This is why the procedure is ideally repeated many times and the results are averaged over these repeated Monte Carlo runs. This approach is available in Stata and R, where it presents results on the mean/risk difference scale [27]. Details on the modeling of counterfactual outcomes, and estimation of the direct and indirect effects using Monte Carlo simulations are presented in supplement S4: The estimation algorithm.

Another quantity of interest is the proportion mediated (PM). This measure assesses to what extent the TTE on the outcome operates through the mediator. The PM is defined as the ratio of IE to the total treatment effect: PM = IE/TTE [27].

Statistical analyses are performed using the *medeff* command integrated in the analytical software Stata version 16.1: StataCorp, College Station, TX, USA [31].

## 3. Results

### 3.1. Mediator and Outcome Models

The M model estimates the effect of treatment on the success probability of VMAR, controlling for the baseline covariates age, focal adhesion, and ERM status, and including a treatment by age interaction.

The Y models estimate the effect of treatment on the success probability of VFQ-I, VA-I, and VF-I, adjusted for the binary variable VMAR and relevant baseline covariates C (supplement S5: Logistic regression models). The goodness-of-fit statistic confirmed the models’ adequacy (supplement S6: Model specifications for mediator and outcome models). There was no evidence of treatment by mediator interaction (*p* = 0.68, VFQ-I; *p* = 0.85, VA-I; *p* = 0.73, VF-I) (S7: Likelihood Ratio Tests for outcome models with vs. without treatment–mediator interaction). We therefore present the results that assume no interaction.

We report the ocriplasmin treatment effect on vision outcomes expressed on the odds ratio scale and on the probability scale in Tables S5.2 and S5.3, respectively, in supplement S5: Logistic regression models.

### 3.2. Causal Mediation Effects

In OASIS, a question of interest is whether treatment with ocriplasmin, compared to sham, leads to better vision improvements at month 24 by increasing the rate of VMAR by day 28, i.e., whether VMAR is a mediator of the ocriplasmin treatment effect. We report this for the total population, and for the subgroup with VMT only. We evaluate if adjustment for a larger number of baseline covariates would change our causal estimates and explore the dynamics of interaction by considering a treatment by mediator interaction.

1. Total population analysis

Table 1 displays the estimated causal effects for the three vision outcomes. The IE for VFQ-I, VA-I, and VF-I is 5.7%, 11.8%, and 5.2%, respectively.

The IE under treatment represents what would happen to the vision outcome if VMAR status were changed for each participant to the extent that it is affected by ocriplasmin for that participant. In particular, the probability for a participant of a VF-I at month 24 increases by 5.2% as a result of the ocriplasmin effect on the participant’s VMAR status.

DE is 8.3%, 12.1%, and 24.1%, respectively. DE captures the effect of ocriplasmin on the vision outcome if the treatment left each participant’s VMAR status unchanged. In particular, the ocriplasmin effect on VF-I at month 24 as a result of its effect through any pathway other than VMAR is 24.1% (i.e., ocriplasmin left each participant’s VMAR status as if each participant was treated with sham).

The PM shows the part of the TTE that is due to the effect of VMAR. It shows that 17.7% of the treatment effect on VF-I is mediated by the treatment effect on VMAR (5.2/29.3). The PM for VA-I is higher at 48.9%. Changing the VMAR status of a participant who received ocriplasmin $(Y(1,M\left(1\right))\;$to the VMAR status that would have been in the sham regimen for that participant $\left(Y(1,M\left(0\right)\right)$ decreases the probability of VA-I with 11.8%.

A visualization of the TTE break down into direct and indirect estimands for outcome VF-I is shown in supplement S3: Breakdown of total treatment effect (Figure S3).

2. Subgroup analyses

The IE estimates in the VMT subgroup (no concomitant FTMH) are significant for all vision outcomes, ranging between 6.5% and 10.7% (Table 2). The average TTE is significant for the outcome VF-I, not for VA-I and VFQ-I. The PM for VFQ-I and VA-I shows wide confidence intervals (CIs).

3. Additional analyses

Table 3 reports a mediation analysis on the same participants but extending the list of baseline covariates, interactions, and higher-order terms to include the anatomic, vision, and demographic covariates. Compared to the primary analysis (Table 1), we conclude that the extended covariate adjustment generally leads to similar effect sizes, and more precision for the IE estimates for VF-I.

## 4. Discussion

We investigated the potential causal pathways linking ocriplasmin, VMAR, and vision outcomes by applying a causal inference approach to mediation analysis. Our results indicate that the effect of ocriplasmin on VA-I was to a large extent the result of VMAR, while the improvement in PROs was moderately explained by VMAR. These findings provide additional insights into the role of VMAR as a biomarker for longer-term functional or PROs. To the best of our knowledge, no causal mediation analysis has yet been conducted to investigate the effect of treatment on outcomes, mediated through an intermediate variable (mediator), in either vitreomacular traction or other eye disorders. The specific aim of our analysis was to disentangle the causal effect of the mediator—VMAR (indirect effect)—from the effects of other pathways, such as treatment and additional mechanisms (direct effect), on vision outcomes. Previous subgroup analyses compared the effect of VMA status on outcomes, treating the post-treatment covariate VMAR as if it were randomly assigned [9,32]. Since indirect effects involve drawing conclusions about the mediator’s impact on the outcome, it is essential to control for confounding between the mediator and the outcome. The strength of this study’s methodology lies in its ability to adjust for (measured) confounding in the mediator–outcome relationship. Additionally, our analysis adjusted for baseline covariates such as visual acuity and visual functioning, which are also the outcome measures of interest. This study’s methodology, therefore, provides deeper insights into mediation than a simple subgroup analysis.

We found that ocriplasmin was associated with significantly increased improvements in VA-I, VFQ-I, and VF-I. The IE estimates of ocriplasmin on VFQ-I and VA-I were positive and significant with a sizeable PM, 40% and 49%, respectively. This provides evidence that the effect of ocriplasmin on functional and PROs was partially mediated through VMAR, consistent with the pathophysiology of VMT that release of VMA decreases the tractional forces on the macula, reduces foveal deformation, and lessens visual dysfunction [2,33]. The relationship between ocriplasmin and VF-I, explained by a treatment-related release in VMA, was not significant. This may potentially be attributed to measurement properties of VF-I and requires further investigation. Conversely, the DE analyses, which controlled for VMAR, showed that the treatment effect on VA-I and VF-I (but not VFQ-I) were significant with a smaller magnitude of effects than its respective total effect measures. This suggest that ocriplasmin directly, on top of the VMAR mechanism, affects VA and VF positively. From a clinical perspective, it is plausible that VA-I is more sensitive to treatment effects compared to the patient-reported outcomes VFQ-I and VF-I. This is because VA-I has a more direct correlation with the anatomical endpoint of VMAR. Many factors can influence a patient’s perceived quality of life (QoL). Visual acuity is just one measure of visual function, and many participants may have primarily experienced distortion rather than blurred vision, which can also significantly impact day-to-day visual functioning. Even if visual acuity improves as a result of traction resolution, this does not necessarily translate into an enhanced QoL. Conversely, even if visual acuity remains unchanged, patients might still experience visual benefits if traction resolution reduces their distortion. Moreover, fellow eye visual function can also factor into patient questionnaires that assess overall vision. In addition, the VFQ-25 instrument, used to assess vision-related QoL, is known to suffer from ceiling effects and may have been insensitive to changes in the majority of participants, potentially diluting the observed treatment effect [34]. Finally, the impact of VMAR measurements may vary depending on whether we are examining isolated VMT or VMT associated with a macular hole. In VMT, the primary goal is to relieve traction, while in cases involving a macular hole, the objective is to close the hole. The VMT subgroup analysis (Table 2) demonstrates that the overall treatment effects on VA-I and VFQ-I are similar.

Considering the concern for bias due to model misspecification, we built the logistic regression models using the general strategy by Collett [35] to decide on which baseline variables were associated with the outcomes and to investigate if model extensions with quadratic and/or interaction terms were needed. Additionally, statistical model assumptions were verified using Hosmer–Lemeshow goodness-of-fit assessment. To control for potential confounding, we further adjusted each model to include all available baseline anatomic, vision, and demographic covariates. This extended covariate adjustment had a limited impact on the causal effect sizes, but improved the precision of the IE for VF-I. A richer set of baseline covariates strengthens the plausibility of the no unmeasured confounding assumption of the mediator–outcome relationship [13,16].

In observational studies, strong assumptions are needed to interpret (in)direct effect estimates as causal [21,27]. In randomized trials, as in OASIS, it is appropriate to assume that the treatment–mediator and treatment–outcome effects are not confounded by baseline variables because of the random allocation of the treatment [20]. However, when there are mediator–outcome confounding variables that are unmeasured or for which control has not been made, estimates of (in)direct effects may be biased and conclusions cannot be drawn about the effects of the mediator on the outcome [21,27]. Based on our additional analysis with extended covariate adjustment, we believe control for common causes of the mediator and outcome was reasonably well considered. Finally, the validity of mediation analyses also depends on the core assumption that there is no effect of the treatment that itself confounds the mediator–outcome relationship. This assumption requires that there is nothing on the pathway from the treatment to the mediator that also affects the outcome. This is not problematic if all common causes are baseline covariates, but if a confounder for both the mediator and outcome is also affected by treatment, these post-treatment confounders may act as mediator. This analysis did not consider the possibility of post-treatment confounders. This may be a plausible assumption as the mediator occurs shortly (28 days) between treatment and assessment of the mediator.

Our primary analysis assumes no interaction between the effects of the treatment and the mediator on the outcome. We relaxed this assumption by adding a treatment–mediator interaction to potentially describe the dynamics of mediation. We observed that the causal effect estimates remain largely similar (supplement S8: Mediation effect estimates in the presence of treatment–mediator interaction) and conclude that the no-interaction assumption is appropriate.

In the VMT subgroup, ocriplasmin was associated with significantly increased improvements in the VF-I only. This finding could be due to the small sample size of the VMT subgroup dataset. The relationship between treatment and VA-I substantially differed in this subgroup, with a less pronounced treatment effect. Specifically, the magnitude of the total effect on VA-I was approximately 10% smaller compared to the overall population, which may indicate the impact of baseline mean VA (57.8 vs. 65.9 letters in the subjects with FTMH vs. VMT only, respectively). The indirect effects on all vision outcomes were statistically significant, implying an effect of mediation through VMAR. In particular, the proportions mediated were larger for VFQ-I and VA-I (49.5% and 71.7%, respectively) compared to VF-I (20.7%). The PM is expressed as a ratio of indirect and total treatment effect, which may explain the high magnitude of this measure in the case of VA-I (71.70%). Because there can be considerable uncertainty around the PM, especially in small samples, keeping the focus on the indirect and direct effects of interest is recommended [20].

The use of surrogate endpoints (SEPs) has become more common in the licensing of new health technologies; however, for HTA agencies, such evidence increases the uncertainty in their coverage decisions [36]. Indeed, given that HTA agencies focus on a longer-term perspective and seek to assess clinical effectiveness and cost-effectiveness, versus safety and shorter-term efficacy, their considerations on the acceptability of SEPs may differ from those of regulators [6]. To ensure the surrogate is adequate, several HTA agencies endorse the principles of the EUnetHTA guideline on SEPs. However, a recent overview of HTA guidelines noted the current lack of consensus on the minimum criteria to establish the validity of SEPs [6,36,37]. Regulators have considered VMAR as the primary endpoint for assessing the effect of treatment in VMT. This raises a question about surrogacy, i.e., whether this anatomic biomarker is a consistent surrogate such that data about the effect of ocriplasmin on VMAR can be used to make decisions about the direction of the treatment effect on vision outcomes [27]. Following the *BEST* guidelines, VMAR can be deemed a reasonably likely SEP as it is supported by a strong mechanistic and/or epidemiological rationale [38]. Indeed, there is epidemiological evidence that the release of traction is associated with improved VA outcomes. More specifically, in a meta-analysis of twelve observational studies on the natural history of VMT, patients who experienced a spontaneous release of VMT had an average improvement of approximately two lines in VA, compared to those without traction release [39]. So, using VMAR as a candidate mediator to investigate the causal mechanism of how ocriplasmin affects vision outcomes makes sense. Further, the *BEST* guidelines state that the treatment effect on the SEP is expected to be correlated with an endpoint intended to assess clinical benefit [38]. At this point in time, however, there are insufficient clinical data to demonstrate that VMAR is a validated SEP in VMT; hence, we suggest additional research on this topic [5]. The IE approach, which relies on the concepts of mediation, is one way to assessing surrogacy. However, the criterion used to assess whether a surrogate is good (the PM is large) does not guarantee that the surrogate is consistent because a good surrogate does not need to mediate the effect. The meta-analytic approach is an alternative method to assess surrogacy and employs multiple studies or subgroups [6,27,36,37]. A meta-analysis based on individual participant data from several RCTs in VMT has previously confirmed that vision outcomes varied across subgroups and hence could be used to assess this question of surrogacy [25]. Ultimately, the forthcoming joint HTA clinical assessment may provide the opportunity for implementation of a harmonized approach to the validation of SEPs across Europe [36].

Apart from a scientific interest in studying the mechanisms, pathways, and intermediate variables whereby a treatment affects an outcome, there may be practical consequences for why one would want insights into the phenomenon of mediation. More specifically, intervening on the mediator may be achievable with alternative interventions that are cheaper or with potentially fewer side effects [40]. Also, if a treatment has demonstrated a beneficial effect on average, it may be attractive to refine the intervention to increase its effect size, for example, by modifying components of the intervention that target a particular mechanism for the outcome. Clearly, it is more efficient to know in advance whether, and to what extent, the targeted mechanism is an important pathway from the intervention to the outcome. If the mechanism explains a large portion of the effect, then refining the intervention further to target this mechanism may be desirable. In the case of pharmacological vitreolysis, assuming ocriplasmin variants are discovered with higher therapeutic activity, understanding the relative importance of VMAR vs. any other mechanisms is critical to further functional and therapeutic research [41].

Analyses of randomized trials frequently supplement the primary intention-to-treat analysis with analyses aimed at a better understanding of the treatment mechanism. Mediation analysis seeks a more in-depth understanding by breaking down the intention-to-treat effect into a direct and indirect effect, mediated by given intermediaries [42]. Applying causal mediation in other retinal disorders such as neovascular age-related macular disease (nAMD) can have a massive impact, especially since the advent of advanced imaging technology and artificial intelligence have stimulated the investigation of numerous biomarkers as an intermediary or surrogate for long-term vision outcomes [43,44,45,46,47]. Myriad new sustained therapies are being developed to ease the treatment burden without compromising vision. Studying the causal pathways of how these sustained treatments affect outcomes represents an important vision health topic.

In our analysis, we demonstrate that ocriplasmin affects outcomes; however, proposing VMAR as the sole mechanism to explain how ocriplasmin affects vision, especially for the patient-centered outcomes (VFQ-I and VF-I), may have limitations. We therefore suggest further research into alternative mechanisms. One approach could be to explore how to deal with intercurrent events such as vitrectomy, without having to revert to a binary outcome as this leaves a lot of the information out of the analysis. Indeed, in this analysis, participants undergoing a vitrectomy are automatically considered as an outcome failure. The resulting estimand is consistent with the composite endpoint strategy [48]. An additional mediation analysis considered the continuous variables VA and VFQ-25cs, regardless of vitrectomy, as the outcomes of interest. This analysis is consistent with the treatment policy strategy, whereby vitrectomy is viewed as part of the treatment algorithm, thereby changing the estimand [48]. Alternatively, one could estimate what the treatment effect would be had there been no rescue therapy or had the use of rescue therapy been similar in both treatment arms. In those two cases, vitrectomy is itself viewed as a mediator. The issue of how to deal with rescue therapy is topical and important to search for a methodology transferable to other therapeutic areas. Finally, another investigation could be to measure VMAR using a continuous intermediary and explore if/how this mediator definition impacts the mediated effects.

## 5. Conclusions

Clinical trials stop short of explaining how a causal relationship happens. A mediation analysis quantifies the extent to which the effect of one variable on another is mediated by some possible intermediate variable(s). We quantitatively investigated the causal mechanism whereby ocriplasmin affects patient-relevant outcomes by exerting an effect on the intermediate variable VMAR, being the primary endpoint in the OASIS clinical trial. We found that the effect of ocriplasmin on VA was to a large extent the result of VMAR, while the improvement in PROs was only partially the result of this intermediary variable.

## Figures and Tables

**Table 1:** Causal effect estimates for the binary vision outcomes—total study population.

	VFQ-I (%) *	VA-I (%) *	VF-I (%) *
Average IE(1) † [95% CI]	5.7 ‡ [1.16, 10.86]	11.8 ‡ [4.99, 19.41]	5.2 [−0.31, 11.31]
Average DE(0) † [95% CI]	8.3 [−3.30, 19.53]	12.1 ‡ [1.53, 22.95]	24.1 ‡ [11.58, 36.56]
Average TTE [95% CI]	13.9 ‡ [2.61, 24.23]	23.9 ‡ [12.15, 34.97]	29.3 ‡ [17.81, 40.20]
Proportion Mediated IE(1)/TTE	40.0 [22.75, 167.28]	48.9 [33.67, 96.77]	17.7 [12.98, 29.28]

* Risk difference at month 24; † *IE* (1): indirect effect under treatment condition, ocriplasmin (*t* = 1); † *DE* (0): direct effect under control condition, sham (*t* = 0); *TTE*: total treatment effect (difference in observed % of ocriplasmin-treated participants with a vision response vs. sham-treated participants); ‡ results with significant values at *p* < 0.05. VFQ-I: visual function questionnaire improvement; VA-I: visual acuity improvement; VF-I: visual function improvement.

**Table 2:** Causal effect estimates for the binary vision outcomes—VMT subgroup.

	VFQ-I (%) *	VA-I (%) *	VF-I (%) *
Average IE(1) † [95% CI]	7.5 ‡ [2.19, 13.21]	10.7 ‡ [3.71, 18.42]	6.5 ‡ [0.14, 14.03]
Average DE(0) † [95% CI]	5.15 [−11.18, 21.29]	3.37 [−13.49, 21.15]	24.9 ‡ [8.90, 40.68]
Average TTE [95% CI]	12.6 [−6.50, 29.12]	14.1 [−2.10, 29.77]	31.5 ‡ [16.07, 45.63]
Proportion Mediated IE(1)/TTE	49.5 [−414.38, 496.92]	71.7 [−387.39, 497.93]	20.7 [14.37, 40.81]

* Risk difference at month 24; † IE (1): indirect effect under treatment condition, ocriplasmin (t = 1); † DE (0): direct effect under control condition, sham (t = 0); TTE: total treatment effect (difference in observed % of ocriplasmin-treated participants with a vision response vs. sham-treated participants); ‡ results with significant values at *p* < 0.05. VFQ-I: visual function questionnaire improvement; VA-I: visual acuity improvement; VF-I: visual function improvement.

**Table 3:** Causal effect estimates for the binary vision outcomes—extended covariate adjustment.

	VFQ-I (%) *	VA-I (%) *	VF-I (%) *
Average IE(1) † [95% CI]	4.3 [−0.80, 9.76]	10.5 ‡ [3.64, 18.16]	5.4 ‡ [1.40, 10.39]
Average DE(0) † [95% CI]	10.7 [−0.98, 22.06]	14.4 ‡ [3.20, 25.57]	23.1 ‡ [10.56, 35.21]
Average TTE [95% CI]	15.0 ‡ [3.99, 24.96]	24.9 ‡ [13.19, 35.86]	28.5 ‡ [15.50, 40.30]
Proportion Mediated IE(1)/TTE	28.3 [16.85, 99.1]	42.0 [29.26, 79.57]	18.8 [13.43, 34.93]

* Risk difference at month 24; † *IE* (1): indirect effect under treatment condition, ocriplasmin (*t* = 1); † *DE* (0): direct effect under control condition, sham (*t* = 0); *TTE*: total treatment effect (difference in observed % of ocriplasmin-treated participants with a vision-response vs. sham-treated participants); ‡ results with significant values at *p* < 0.05. VFQ-I: visual function questionnaire improvement; VA-I: visual acuity improvement; VF-I: visual function improvement.

## Data Availability

Restrictions apply to the availability of these data. Data were obtained from Oxurion and are available from the corresponding author with the permission of Oxurion.

## Supplementary Materials

The following supporting information can be downloaded at: https://www.mdpi.com/article/10.3390/jmahp12040022/s1, S1: OASIS study characteristics; S2: Confounding assumptions for mediation analysis; S3: Breakdown of total treatment effect; S4: Estimation algorithm; S5: Logistic regression models; S6: Model specifications for mediator and outcome models; S7: Likelihood Ratio Tests for outcome models with vs. without treatment–mediator interaction; S8: Mediation effect estimates in the presence of treatment–mediator interaction.
