# Supplementary material for: A Causal Inference Approach to Mediation Analysis in Vitreomacular Traction: How Much Does Traction Resolution Mediate Functional Outcomes?
Source: J Mark Access Health Policy. 2024 Sep 30;12(4):280–93. doi: 10.3390/jmahp12040022 (PMC11503358; doi:10.3390/jmahp12040022)
Supplement: Supplementary file 1 [file jmahp-12-00022-s001.zip › S1 OASIS study characteristics.pdf]

S1: OASIS study characteristics

The trial sample size was 220 participants (146 ocriplasmin, 74 sham), all enrolled in the United States between November 2011 and October 2012. Two participants (1 in each treatment group) withdrew consent and did not attend any of the post-injection visits.

Table S1: Key baseline patient and ocular characteristics, primary and secondary outcomes

	Sham (N=73)	Ocriplasmin (N=145)
**Baseline demographics**		
Sex, n (%)		
Male	28 (38.4)	43 (29.7)
Race, n (%)		
White	64 (87.7)	131 (90.3)
Black	5 (6.8)	13 (9.0)
Other	4 (5.5)	1 (0.7)
Ethnicity, n (%)		
Non-Hispanic	69 (94.5)	133 (91.7)
Hispanic	4 (5.5)	12 (8.3)
Age, years		
mean, SD	68.5 (11.01)	69.4 (10.02)
Min, Max	39, 89	38, 94
**Baseline ocular status, n (%)**		
Confirmed VMA on SD OCT	72 (98.6)	139 (95.9)
VMA diameter		
Focal, ≤ 1500 µm	62 (84.9)	128 (88.3)
Broad, > 1500 µm	8 (11.0)	8 (5.5)
Missing	3 (4.1)	9 (6.2)
Macular hole present	26 (35.6)	50 (34.5)
Largest of minimum macular hole width		
≤ 250 µm	11 (42.3)	23 (46.0)
> 250 – 400 µm	11 (42.3)	17 (34.0)
> 400 µm	4 (15.4)	10 (20.0)
ERM present	17 (23.3)	33 (22.8)
Phakic lens	51 (69.9)	106 (73.1)
**Functional outcomes**		
BCVA (ETDRS letters), n	73	144
mean (SD)	62.4 (11.05)	63.5 (8.89)
Visual Function Questionnaire, composite score, n	73	145
mean (SD)	81.2 (13.87)	77.7 (14.53)
**Primary efficacy endpoint, n (%)**		
VMA resolution at day 28[*]	*5 (6.2)*[§]	*62 (41.7)*[§]
**Secondary efficacy endpoints, n (%)**		
Improvement in BCVA at month 24[†]	29 (39.1)	73 (50.5)[^]
Improvement in VFQ-25 composite score at month 24[‡]	*22 (26.9)*[§]	*74 (51.4)*[§]
Vitrectomy prior to month 24	32 (43.0)	48 (33.0)
**Follow-up, n (%)**		
Completed 2 years[#]	51 (68.9)	108 (74.0)

Abbreviations: BCVA, best corrected visual acuity; ERM, epiretinal membrane; ETDRS, Early Treatment Diabetic Retinopathy Study (testing protocol); FTMH, full-thickness macular hole; SD, standard deviation; SD OCT, spectral-domain optical coherence tomography; VMA: vitreomacular adhesion.

[*] Without creation of an anatomical defect, post-resolution vitrectomy considered a failure.

[§] Statistically significant.

[^] One ocriplasmin-treated participant had no baseline assessment of visual acuity.

[†] ≥2 lines (10 ETDRS letters) in BCVA from baseline, *irrespective* of vitrectomy.

[‡] ≥5 points improvement in VFQ-25 composite score from baseline, *irrespective* of vitrectomy.

[#] Most of the demographics and baseline ocular characteristics were similar between those who completed (C) the study vs. the non-completers (NC). However, in the C compared with the NC, subjects were younger (<65 years, 36.5% and 27.1%, resp.), presence of FTMH was higher (39.9% and 24.3%, resp.), central retinal thickness was thinner (median central retinal thickness, 119.0µm and 241.5 µm, resp.), presence of subretinal fluid was higher (65.5% and 52.9%, resp.), more subjects were phakic (76.4% and 62.9%, resp.), and the proportion of subjects with definite site(s) of incomplete inner segment/outer segment (IS/OS) band at the fovea and incomplete external limiting membrane (ELM) at the fovea were higher (definite site[s] of incomplete IS/OS band at the fovea: 57.4% and 47.1%, resp.; definite site[s] of incomplete ELM at the fovea: 51.4% and 40.0%, resp.). The LOCF method for imputation of missing data was used for primary and secondary endpoints evaluation, except for the number of vitrectomies endpoint which used observed cases.
