# Supplementary material for: A Causal Inference Approach to Mediation Analysis in Vitreomacular Traction: How Much Does Traction Resolution Mediate Functional Outcomes?
Source: J Mark Access Health Policy. 2024 Sep 30;12(4):280–93. doi: 10.3390/jmahp12040022 (PMC11503358; doi:10.3390/jmahp12040022)
Supplement: Supplementary file 1 [file jmahp-12-00022-s001.zip › S2 Confounding assumptions for mediation analysis.pdf]

# S2: Confounding assumptions for mediation analysis

For the direct and indirect effect estimates to have a causal interpretation, control must be made for all relevant confounding variables. Four assumptions need to be satisfied to estimate the direct and causal mediation effects:

1. *No unmeasured confounding of the treatment – outcome relationship*, given the covariates *C*, i.e., control (adjust) for common causes of the treatment and outcome.

   → Due to the randomized design of the OASIS study, this assumption is automatically satisfied.

2. *No unmeasured confounding of the mediator – outcome relationship*, given the covariates *C* and treatment $T$*, i.e.,* control (adjust) for common causes of the mediator $M$ and the outcome $Y$. Because with direct and indirect effects we are drawing conclusions about the effects of the mediator on the outcome, control must be made for mediator – outcome confounding.

   → Even with randomized treatment this assumption might not be satisfied. Ignorability of the mediator is problematic and often a violated assumption.

3. *No unmeasured confounding of the treatment – mediator relationship*, given the covariates *C*, i.e., given the measured covariates *C*, control for variables that cause both the level of treatment and the level of the mediator. This assumption must be satisfied because mediation analysis is essentially about the exposure changing the mediator $M$ and that change in the mediator $M$ exerting a change in the outcome $Y$. To isolate the effect of the exposure $T$ on the mediator $M$, we need to control for confounding of that relationship.

   → Due to the randomized design of the OASIS study, this assumption is automatically satisfied.

4. *No mediator – outcome confounder that is affected by treatment*, i.e., none of the common causes of the mediator $M$ and outcome $Y$ are themselves affected by the exposure $T$. It requires that there is nothing on the pathway from the exposure to the mediator that also affects the outcome. This may be more plausible if the mediator occurs shortly after the exposure (as in the current analysis with 28 days between exposure and assessment of mediator outcome). This assumption is not problematic if all common causes are baseline covariates, but if you have a confounder for both *M* and *Y* that is also affected by *T*, these post-treatment confounders may also act as mediator.

   → Randomization of treatment rules out the problem of treatment-outcome and treatment-mediator confounding but does not guarantee that the assumption of no confounding of mediator-outcome relationship holds. This is because even if the treatment is randomized, the mediator generally will not be.
