# Supplementary material for: A Causal Inference Approach to Mediation Analysis in Vitreomacular Traction: How Much Does Traction Resolution Mediate Functional Outcomes?
Source: J Mark Access Health Policy. 2024 Sep 30;12(4):280–93. doi: 10.3390/jmahp12040022 (PMC11503358; doi:10.3390/jmahp12040022)
Supplement: Supplementary file 1 [file jmahp-12-00022-s001.zip › S3 Breakdown total treatment effect.pdf]

S3: Breakdown of total treatment effect

Using ***counterfactual definitions, and with a binary treatment***, a total treatment effect can be broken down into a direct and indirect effect as follows:

$TTE_i = Y_i(1) - Y_i(0)$

$= Y_i(1, M_i(1)) - Y_i(0, M_i(0))$ [consistency assumption]

$= Y_i(1, M_i(1)) - Y_i(1, M_i(0)) + Y_i(1, M_i(0)) - Y_i(0, M_i(0))$ [add and subtract the $Y_i(1, M_i(0))$term]

$= [Y_i(1, M_i(1)) - Y_i(1, M_i(0))] + [Y_i(1, M_i(0)) - Y_i(0, M_i(0))]$

$= IE_i(1) + DE_i(0)$

= natural indirect effect + natural direct effect

These effects are defined in terms of counterfactuals, so neither the definition nor the breakdown of the total treatment effect in natural direct and indirect effects presume any specific model or function form, or assumption about interaction.

Note that the term *natural* indirect effect called by Pearl [28] is termed *total* indirect effect by Robins [47].

An alternative break down can be obtained by adding and subtracting the term $Y_i(0, M_i(1))$

$TTE_i = Y_i(1) - Y_i(0)$

$= Y_i(1, M_i(1)) - Y_i(0, M_i(0))$ [consistency assumption]

$= Y_i(1, M_i(1)) - Y_i(0, M_i(1)) + Y_i(0, M_i(1)) - Y_i(0, M_i(0))$ [add and subtract the $Y_i(0, M_i(1))$term]

$= [Y_i(1, M_i(1)) - Y_i(0, M_i(1))] + [Y_i(0, M_i(1)) - Y_i(0, M_i(0))]$

$= DE_i(1) + IE_i(0)$

= natural direct effect + natural indirect effect

Note that the term *natural* indirect effect and *natural* direct effect called by Pearl [46] is termed *pure* indirect effect and *pure/total* direct effect by Robins [29].

Focusing on ***population averages***, the average total effect of treatment $\tilde{\tau}$ can be broken donw into an average indirect effect (AIE) and an average direct effect (ADE) in the following matter [13].

$$\tilde{\tau} \equiv E(Y_i(1, M_i(1)) - E(Y_i(0, M_i(0)))$$

$$= \underbrace{E(Y_i(1, M_i(1)) - E[Y_i(1, M_i(0)))}_{} + \underbrace{E(Y_i(1, M_i(0)) - E[Y_i(0, M_i(0)))}_{}$$

$$= \quad AIE(1) \quad + \quad ADE(0)$$

$$\tilde{\tau} \equiv E(Y_i(1, M_i(1))) - E(Y_i(0, M_i(0)))$$

$$= \underbrace{E(Y_i(1, M_i(1)) - E(Y_i(0, M_i(1)))}_{} + \underbrace{E(Y_i(0, M_i(1)) - E[Y_i(0, M_i(0)))}_{}$$

$$= \quad ADE(1) \quad + \quad AIE(0)$$

If we make the no-interaction assumption that ADE and AIE do not vary as functions of treatment status, meaning AIE = AIE(1) = AIE(0) and ADE = ADE(1) = ADE(0), then the ADE and AIE sum to the average total causal effect $\tilde{\tau}$ = AIE + ADE.

Figure A visually presents the causal effect estimates for the outcome VF-I

Figure S3: Estimated causal quantities: average total treatment, indirect and direct effects for outcome VF-I

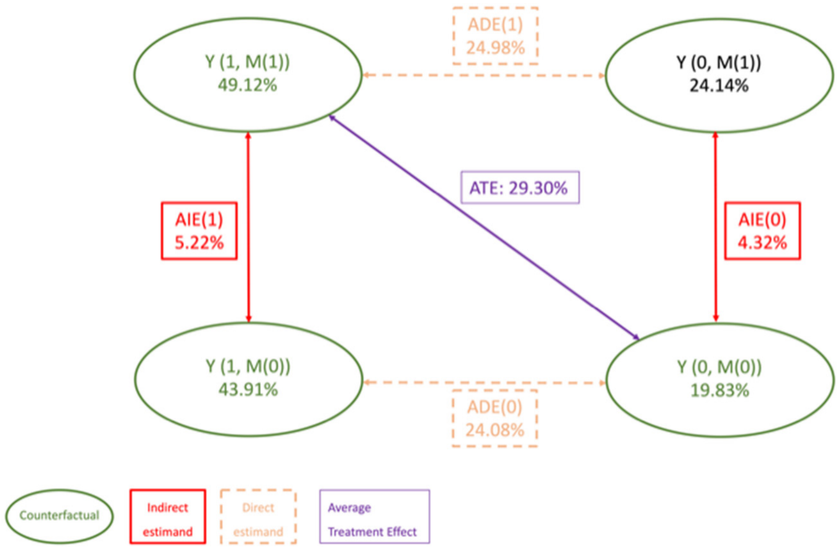

Average indirect effect (t=1) $= E(Y_i(1, M_i(1))) - E(Y_i(1, M_i(0)))$
$= 49.12\% - 43.91\%$
AIE(1) $= 5.22\%$

Average direct effect (t=0) $= E(Y_i(1, M_i(0))) - E(Y_i(0, M_i(0)))$
$= 43.91\% - 19.83\%$
ADE(0) $= 24.08\%$

Average indirect effect (t=0) $= E(Y_i(0, M_i(1))) - E(Y_i(0, M_i(0)))$
$= 24.14\% - 19.83\%$
AIE(0) $= 4.32\%$

Average direct effect (t=1) $= E(Y_i(1, M_i(1))) - E(Y_i(0, M_i(1)))$
$= 49.12\% - 24.14\%$
ADE(1) $= 24.98\%$

Average total treatment effect **= 29.30%**
AIE(1) + ADE(0) $= 5.22\% + 24.08\%$
AIE(0) + ADE(1) $= 4.32\% + 24.98\%$

[13] Keele, L.; Tingley, D.; Yamamoto, T. Identifying Mechanisms behind Policy Interventions via Causal mediation Analysis. *J. Policy Anal. Manag.* **2015**, *34*, 937–963. https://doi.org/10.1002/pam.21853.
[28] Pearl, J. Direct and Indirect Effects. In Proceedings of the Seventh Conference on Uncertainty in Artificial Intelligence, San Francisco, CA, USA, 13–15 July 1991; Morgan Kaufmann: Cambridge, MA, USA, 2001; pp. 411–420. Available online: https://ftp.cs.ucla.edu/pub/stat_ser/R273-U.pdf (accessed on 15 February 2024).
[29] Robins, J.M. Semantics of Causal DAG Models and the Identification of Direct and Indirect Effects. Highly Structured Stochastic Systems; Oxford University Press: New York, NY, USA, 2003; pp. 70–82. https://doi.org/10.1093/oso/9780198510550.003.0007.
