# Supplementary material for: A Causal Inference Approach to Mediation Analysis in Vitreomacular Traction: How Much Does Traction Resolution Mediate Functional Outcomes?
Source: J Mark Access Health Policy. 2024 Sep 30;12(4):280–93. doi: 10.3390/jmahp12040022 (PMC11503358; doi:10.3390/jmahp12040022)
Supplement: Supplementary file 1 [file jmahp-12-00022-s001.zip › S4 Estimation algorithm.pdf]

S4: Estimation algorithm

Modeling the counterfactual outcomes

The estimation of average direct and indirect effects is based on the so-called mediation formula. This involves first predicting each individual participant's counterfactual mediator $M_i(t)$ at each treatment level *t*=0,1, using a regression model for the mediator in function of treatment and covariates. Here, `stochastic' predictions are used, meaning that the fitted probabilities delivered by the regression model are subsequently used to generate counterfactual mediator values for each participant. Next, counterfactual outcomes are predicted for each participant at each treatment level, using a regression model for the outcome in function of mediator, treatment, and covariates.

Separate statistical models are defined for the M and Y to describe the relationship between the probability of a successful response and the covariates as independent variables. Given the binary nature of *M* and *Y,* we specify the following logistic regression models:

$$\begin{cases} \text{logit}(\text{P}(M_i = 1)) = \alpha_1 + \beta_1 T_i + \xi_1 C_i & (1) \\ \text{logit}(\text{P}(Y_i = 1)) = \alpha_2 + \beta_2 T_i + \gamma M_i + \xi_2 C_i & (2) \end{cases}$$

The mediator model (1) predicts whether participant *i* experiences VMAR at day 28 based on the participant's treatment T_i (ocriplasmin or sham) and relevant baseline covariates C_i. The outcome model (2) predicts whether participant *i* experiences e.g. VF-I at month 24 based on the participant's treatment T_i, mediator M_i status and relevant baseline covariates C_i. To estimate (in)direct effects, control must be made for all variables that confound the relationships between M_i and Y_i. This explains the inclusion of covariates C_i.

The logistic regression models for M and Y were built using the general strategy for model selection by Collet (Collett 2003), to decide on the inclusion or exclusion of baseline covariates C_i, the incorporation of higher order terms (nonlinearities) and/or interaction terms between covariates C_i and T_i or C_i and M_i (Table B). To determine how well the selected model fitted the observed data, goodness-of-fit was assessed using the Hosmer-Lemeshow statistic.

In model (2) we assume there is no interaction between T and $M.$ However, causal effects may vary as a function of treatment with ocriplasmin vs. sham. To describe how mediation effects may differ depending on treatment, we added an interaction term $T_i * M_i$ resulting in outcome model

$$\text{logit}(\text{P}(Y_i = 1)) = \alpha_3 + \beta_3 T_i + \gamma M_i + \lambda T_i * M_i + \xi_2 C_i \quad (3)$$

A likelihood ratio (LR) test is performed comparing models (2) and (3) to assess whether the interaction term is required.

To illustrate the generation of the counterfactual outcomes for each participant and derive the causal effects of interest from those, we continue with the outcome model (2) without interaction term.

The IE are generated by the following models for the counterfactual outcomes:

For $IE_i(1)$, the difference between the counterfactuals $Y_i(1, M_i(1))$ and $Y_i(1, M_i(0))$ (*t*=1) follows from:

$$\begin{cases} \text{logit P}(Y_i(1, M_i(1) = 1) = \alpha_2 + \beta_2 + \gamma M_i(1) + \xi_2 C_i & (4) \\ \text{logit P}(Y_i(1, M_i(0) = 1) = \alpha_2 + \beta_2 + \gamma M_i(0) + \xi_2 C_i & (5) \end{cases}$$

and for $IE_i(0)$, the difference between the counterfactuals $Y_i(0, M_i(1))$ and $Y_i(0, M_i(0))$ (*t*=0) follows from:

$$\begin{cases} \text{logit P}(Y_i(0, M_i(1) = 1) & = \alpha_2 + \gamma M_i(1) + \xi_2 C_i \quad (6) \\ \text{logit P}(Y_i(0, M_i(0) = 1) & = \alpha_2 + \gamma M_i(0) + \xi_2 C_i \quad (7) \end{cases}$$

Specifically, from expressions (4)-(7), we obtain the predicted probabilities for the counterfactual outcomes:

$$\begin{cases} \text{P}(Y_i(1, M_i(1)) = 1) = \dfrac{\exp(\alpha_2 + \beta_2 + \gamma M_i(1) + \xi_2 C_i)}{1 + \exp(\alpha_2 + \beta_2 + \gamma M_i(1) + \xi_2 C_i)} \\ \text{P}(Y_i(1, M_i(0)) = 1) = \dfrac{\exp(\alpha_2 + \beta_2 + \gamma M_i(0) + \xi_2 C_i)}{1 + \exp(\alpha_2 + \beta_2 + \gamma M_i(0) + \xi_2 C_i)} \end{cases} \quad (8)$$

and

$$\begin{cases} \text{P}(Y_i(0, M_i(1)) = 1) = \dfrac{\exp(\alpha_2 + \gamma M_i(1) + \xi_2 C_i)}{1 + \exp(\alpha_2 + \gamma M_i(1) + \xi_2 C_i)} \\ \text{P}(Y_i(0, M_i(0)) = 1) = \dfrac{\exp(\alpha_2 + \gamma M_i(0) + \xi_2 C_i)}{1 + \exp(\alpha_2 + \gamma M_i(0) + \xi_2 C_i)} \end{cases} \quad (9)$$

The obtained probabilities for the four counterfactuals for each participant are used in a Monte Carlo approach that repeats these simulations many times.

Monte Carlo simulations to estimate total, direct and indirect effect estimates

The Monte Carlo approach simulates values of the counterfactual outcomes $Y_i(T_i, M_i(T_i))$ used to derive the (in)direct effects per participant, which are then averaged over all study participants. From the different Monte Carlo draws, we obtain different summary statistics of the (in)direct effects, i.e., the point estimate for the mean and its uncertainty estimates from the distribution of effects (Imai, Keele & Tingley 2010).

The Monte Carlo approach consists of different steps:

First, we fit M and Y models to obtain the model parameter estimates $\theta_M = (\alpha_1, \beta_1, \xi_1)$ and $\theta_Y = (\alpha_2, \beta_2, \gamma, \xi_2)$ with their variance and construct the sampling distribution of the maximum likelihood estimators of parameters θ_M and θ_Y assuming the multivariate normal distribution.
Next, the Monte Carlo simulation is started. To account for the uncertainty of the model coefficient estimates, a value is drawn from the sampling distributions of the maximum likelihood estimators of $\theta_M = (\alpha_1, \beta_1, \xi_1)$ and $\theta_Y = (\alpha_2, \beta_2, \gamma, \xi_2)$ at each iteration. Based on this value, probabilities are derived for each participant which are next used to randomly draw the counterfactual outcomes for each participant. The counterfactual outcomes are used to derive the indirect and direct effects per participant, which are then averaged over all study participants.

The algorithm goes as follows(Imai, Keele & Tingley 2010):

1. Set outer loop index to 1, i.e., *k*=1,
2. Draw a random sample of the parameters for the mediator M and outcome Y models from the sampling distribution of the maximum likelihood estimators of $\theta_M^{(k)} = (\alpha_1, \beta_1, \xi_1)$ and $\theta_Y^{(k)} = (\alpha_2, \beta_2, \gamma, \xi_2)$
3. Set inner loop index to 1, i.e., *l*=1,
4. For each participant *i, i*=1,…,n, obtain probability $\text{P}(M_i = 1)$ from model (1) for the actual treatment given, i.e., T_i, and also for the other treatment 1- T_i and draw randomly from a

Bernoulli distribution using these probabilities resulting in binary outcomes $M_i^{(kl)}(0)$ and $M_i^{(kl)}(1)$. Hence, we simulate two counterfactual values of the mediator M, each based on the mediator model, one under the treatment condition and the other under the control condition.

5. For each participant *i, i*=1,…,n, obtain probability $\mathrm{P}(Y_i = 1)$ from (2) for the actual treatment given, i.e., T_i, and also for the other treatment 1- T_i, and further using the mediator values $M_i^{(kl)}(0)$ and $M_i^{(kl)}(1)$ obtained in step 4. Draw randomly from a Bernoulli distribution using these probabilities resulting in binary outcomes $Y_i^{(kl)}(1, M_i^{(kl)}(0))$, $Y_i^{(kl)}(1, M_i^{(kl)}(1))$, $Y_i^{(kl)}(0, M_i^{(kl)}(0))$ and $Y_i^{(kl)}(0, M_i^{(kl)}(1))$. Hence, we simulate the counterfactual outcomes $Y_i(T_i, M_i(T_i))$ given the simulated values of the mediator:
6. Set *l*=*l*+1, and return to 4 until *l*=*L*. If *l*=*L* go to 7.
7. Calculate the average indirect and direct effects
    a. $IE^{(k)}(1) = \frac{1}{nL}\sum_{l=1}^{L}\sum_{i=1}^{n} Y_i^{(kl)}\left(1, M_i^{(kl)}(1)\right) - Y_i^{(kl)}\left(1, M_i^{(kl)}(0)\right)$
    b. $IE^{(k)}(0) = \frac{1}{nL}\sum_{l=1}^{L}\sum_{i=1}^{n} Y_i^{(kl)}\left(0, M_i^{(kl)}(1)\right) - Y_i^{(kl)}\left(0, M_i^{(kl)}(0)\right)$
    c. $DE^{(k)}(1) = \frac{1}{nL}\sum_{l=1}^{L}\sum_{i=1}^{n} Y_i^{(kl)}\left(1, M_i^{(kl)}(1)\right) - Y_i^{(kl)}\left(0, M_i^{(kl)}(1)\right)$
    d. $DE^{(k)}(0) = \frac{1}{nL}\sum_{l=1}^{L}\sum_{i=1}^{n} Y_i^{(kl)}\left(1, M_i^{(kl)}(0)\right) - Y_i^{(kl)}\left(0, M_i^{(kl)}(0)\right)$

    Hence, the values of the counterfactual outcomes $Y_i(T_i, M_i(T_i))$ are averaged across the *L* copies and the *n* participants in the sample to obtain an estimate of the average indirect and direct effects.

    Set *k*=*k*+1, and return to 2 until *k*=*K*. If *k*=*K* stop algorithm.

The number of simulations to run for the approximation of parameter uncertainty was set at 10,000, and default standard errors were calculated. Below table displays a restricted set of patients with observed data and Stata model predictions.

Table S4. A restricted set of patients with observed data and Stata model predictions

Patient number	1	2	3	4	5	…	214	215	216	217	218
OASIS observed values, covariates, treatment mediator and 24 month outcome for VF-I											
Treatment T_i	1	1	1	0	0	…	1	1	0	1	0
Mediator M_i	1	0	1	0	0	…	0	0	0	1	0
Age (centered)	-7.11	1.89	-3.11	8.89	1.89	…	-5.11	19.89	-3.11	-0.11	4.89
ERM absent	1	1	1	0	1	…	0	0	1	1	0
MH present	1	0	1	0	0	…	1	0	1	1	0
Female	1	1	1	1	1	…	1	0	1	0	1
VF-I Y_i	0	1	1	1	0	…	1	1	0	0	0
Simulated probabilities and counterfactual outcomes											
Monte Carlo simulation 1, counterfactual outcomes											
$M_i(1)\ \vert C_i$	0	1	0	0	1	…	0	0	0	0	0
$M_i(0)\ \vert C_i$	0	0	0	0	0	…	0	0	0	0	0
$Y_i(1, M_i(1))\ \vert C_i$						…					
$Y_i(1, M_i(0))\ \vert C_i$						…					
$Y_i(0, M_i(1))\ \vert C_i$						…					
$Y_i(0, M_i(0))\ \vert C_i$						…					
Monte Carlo simulation 1, probabilities* (%)											
$\mathrm{P}(M_i(1)) = 1\ \vert C_i]$						…					
$\mathrm{P}(M_i(0)) = 1\ \vert C_i]$						…					

P[$Y_i(1, M_i(1)) = 1$ | C_i]	34.80	77.12	32.96	38.84	77.12	…	8.99	33.32	32.96	31.32	40.82
P[$Y_i(1, M_i(0)) = 1$ | C_i]	34.80	79.18	32.96	38.84	79.18	…	8.99	33.32	32.96	31.32	40.82
P[$Y_i(0, M_i(1)) = 1$ | C_i]	6.56	30.71	6.07	7.71	30.71	…	1.28	6.16	6.07	5.66	8.31
P[$Y_i(0, M_i(0)) = 1$ | C_i]	6.56	33.33	6.07	7.71	33.33	…	1.28	6.16	6.07	5.66	8.31
Monte Carlo simulation 2, counterfactual outcomes											
$M_i(1)$ | C_i	0	1	0	0	1	…	0	0	0	0	0
$M_i(0)$ | C_i	0	0	0	0	0	…	0	0	0	0	0
$Y_i(1, M_i(1))$ | C_i						…					
$Y_i(1, M_i(0))$ | C_i						…					
$Y_i(0, M_i(1))$ | C_i						…					
$Y_i(0, M_i(0))$ | C_i						…					
Monte Carlo simulation 2, probabilities* (%)											
P($M_i(1)) = 1$ | C_i]						…					
P($M_i(0)) = 1$ | C_i]						…					
P[$Y_i(1, M_i(1)) = 1$ | C_i]	31.20	77.03	27.90	32.49	77.03	…	7.26	38.12	27.90	40.48	36.05
P[$Y_i(1, M_i(0)) = 1$ | C_i]	31.20	77.25	27.90	32.49	77.25	…	7.26	38.12	27.90	40.48	36.05
P[$Y_i(0, M_i(1)) = 1$ | C_i]	5.68	30.84	4.89	7.71	30.71	…	1.03	7.57	4.89	8.29	6.97
P[$Y_i(0, M_i(0)) = 1$ | C_i]	5.68	31.10	4.89	6.01	31.10	…	1.03	7.57	4.89	8.29	6.97
…											

Monte Carlo simulation 10,000, counterfactual outcomes											
$M_i(1)$ | C_i	0	1	0	0	1	…	0	0	0	0	0
$M_i(0)$ | C_i	0	0	0	0	0	…	0	0	0	0	0
$Y_i(1, M_i(1))$ | C_i						…					
$Y_i(1, M_i(0))$ | C_i						…					
$Y_i(0, M_i(1))$ | C_i						…					
$Y_i(0, M_i(0))$ | C_i						…					
Monte Carlo simulation 10,000, probabilities* (%)											
P($M_i(1)) = 1$ | C_i]						…					
P($M_i(0)) = 1$ | C_i]						…					
P[$Y_i(1, M_i(1)) = 1$ | C_i]	22.77	58.34	20.27	31.68	58.34	…	11.06	28.67	20.27	22.87	34.96
P[$Y_i(1, M_i(0)) = 1$ | C_i]	18.65	18.17	21.18	61.27	11.06	…	13.77	28.67	20.27	22.87	34.96
P[$Y_i(0, M_i(1)) = 1$ | C_i]	11.79	38.82	10.33	17.36	38.82	…	6.75	15.41	10.33	11.84	19.59
P[$Y_i(0, M_i(0)) = 1$ | C_i]	11.79	31.82	10.33	17.36	31.82	…	6.75	15.41	10.33	11.84	19.59
**Probabilities averaged over the repeated Monte Carlo runs (%)**											
P($M_i(1)) = 1$ | C_i]						…					
P($M_i(0)) = 1$ | C_i]						…					
P[$Y_i(1, M_i(1)) = 1$ | C_i]	21.2	70.7	18.6	30.5	70.7	…	11.1	34.5	18.6	27.5	33.8
P[$Y_i(1, M_i(0)) = 1$ | C_i]	21.2	54.4	18.6	30.5	54.4	…	11.1	34.5	18.6	27.5	33.8
P[$Y_i(0, M_i(1)) = 1$ | C_i]	6.4	38.4	5.5	10.4	38.4	…	3.1	12.3	5.5	8.8	11.9
P[$Y_i(0, M_i(0)) = 1$ | C_i]	6.4	23.3	5.5	10.4	23.3	…	3.1	12.3	5.5	8.8	11.9

*Probabilities between the different runs change even when M(0) and M(1) do not change between the different runs. This is because the parameters are drawn from their sampling distribution.
