# Supplementary material for: A Causal Inference Approach to Mediation Analysis in Vitreomacular Traction: How Much Does Traction Resolution Mediate Functional Outcomes?
Source: J Mark Access Health Policy. 2024 Sep 30;12(4):280–93. doi: 10.3390/jmahp12040022 (PMC11503358; doi:10.3390/jmahp12040022)
Supplement: Supplementary file 1 [file jmahp-12-00022-s001.zip › S5 Logistic regression models.pdf]

S5: Logistic regression models

Functional forms

We use the following Stata command and functional form for the mediator model *VMA resolution (Med)*:

. logit Med ***i.rt*** *c.age i.focal* ***i.erm rt#c.age****, or*

Treatment with ocriplasmin (*p*<.001) and baseline covariate ERM (*p*=.001) statistically significantly predicted the odds of VMA resolution. The odds of VMA resolution for ocriplasmin-treated participants are 12.26 times the odds for sham-treated participants (95% CI: 4.433 – 33.907). The odds of VMA resolution for participants without ERM are 8.76 times the odds for participants with ERM (95% CI: 2.450 – 31.341). For ocriplasmin-treated participants, a one-year increase in age decreases the odds of VMA resolution by 12% [((0.84*1.04) – 1))*100] (95% CI: 0.709 – 1.090). [a one-unit increase in age yields a change in log odds of (0.041 + (-0.170)) = -0.129 while the exp(-0.129) = 0.8790, the OR for treated participants for a one-unit increase in age).

For the outcome model *VFQ-I*, we use the Stata command and functional form:

. logit VFQ-I i.rt ***i.Med c.va*** *c.va#c.va* ***c.vfq c.vfq#c.vfq i.mh****, or*

VMA resolution (*p*<.05), macular hole (*p*<.01), baseline VA (*p*<.05) and VFQ-25 cs (*p*<.001) statistically significantly predicted the odds of VFQ-improvement. The odds of VFQ-I for participants with VMA resolution are 2.9 times the odds for participants without VMA resolution. The odds of VFQ-I for participants with a macular hole are 0.23 times the odds for participants without a macular hole, meaning we expect an 77% [(0.23 – 1)*100] decrease in the odds of VFQ-I in the presence of a macular hole compared to participants without a macular hole. For a one-unit increase in VA, we expect a 5.2% [((1.06*0.99) – 1)*100)] increase in the odds of VFQ-I (95% CI: 0.990-1.119). For a one-unit increase in the VFQ-25 cs, we expect a 10% decrease [((0.91*1.00) – 1))*100] in the odds of VFQ-I (95% CI: 0.871-0.949).

For the outcome model VA-I, we use the Stata command and functional form:

. logit VA-I ***i.rt i.Med c.age*** *c.age#c.age i.mh* ***i.sex Med#mh****, or*

Treatment (*p*<.05), VMA resolution (*p*<.001), female sex (*p*<.05), age (*p*<.001) and the interaction between VMA resolution and macular hole (*p*<.05) statistically significantly predicted the odds of VA-I. The odds of VA-I for ocriplasmin-treated participants are 2.50 times the odds for sham-treated participants (95% CI: 1.053-5.920). The odds of VA-I for participants with VMA resolution are 5.75 times the odds for participants without VMA resolution (95% CI: 2.149-15.371). The odds of VA-I for female participants are 0.44 the odds for male participants (95% CI: 0.200-0.992). For a one-unit increase in age, we expect an 8.3% [((0.92*0.99) – 1)*100)] decrease in the odds of VA-I (95% CI: 0.875-0.959). The odds of VA-I in participants with macular hole and with VMA resolution are 0.83 (0.14*5.75) times the odds for those without macular hole and without VMA resolution (95% CI: 0.067-10.401), meaning we expect an 17% decrease [0.83-1)*100)] in the odds of VA-I.

For the outcome model VF-I, we use the Stata command and functional form:

. logit VF-I ***i.rt*** *i.Med* ***c.age*** *i.erm* ***i.mh*** *i.sex, or*

Treatment (*p*<.001), macular hole (*p*<.001) and age (*p*<.05) statistically significantly predicted the likelihood of VF-I. The odds of VF-I for ocriplasmin-treated participants are 4.09 times the odds for sham-treated participants (95% CI: 1.849-9.033). The odds of VF-I for participants with a macular hole are 0.15 times the odds for participants without a macular hole (95% CI: 0.069-0.318), meaning we expect an 85% [(0.15 – 1)*100] decrease in the odds of VF-I in the presence of a macular hole compared to participants without a macular hole. For a one-unit increase in age, we expect an 3.9% [(0.96 – 1)*100)] decrease in the odds of VA-I (95% CI: 0.930-0.992). Table B summarizes the logistic regression results for mediator and outcome models.

Table S5.1: Results of the OASIS trial logistic regression models for Mediator and Outcomes

Baseline covariates *C*	Odds Ratio	Robust SE	z-statistic	p-value	[95% Confidence interval]	
			Mediator model VMA resolution (Med)			
Treatment (1=ocriplasmin; 0=sham)	12.26	6.36	4.83	<.001	4.433	33.907
Age*	1.04	0.05	0.79	.430	0.942	1.152
Focal adhesion (1=Yes; 0=No)	3.71	3.65	1.33	.184	0.537	25.601
Epiretinal membrane (1=No; 0=Yes)	8.76	5.70	3.34	.001	2.450	31.341
Treatment#age ocriplasmin	0.84	0.05	-2.92	.003	0.753	0.946
constant	<.01	<.01	-4.42	<.001	0.000	0.040
			Outcome Model Visual Function Questionnaire Improvement (VFQ-I)			
Treatment (1=ocriplasmin; 0=sham)	1.96	0.94	1.40	.161	0.765	5.017
Med, VMA resolution (1=Yes; 0=No)	2.89	1.26	2.43	.015	1.230	6.798
VA*	1.06	0.03	2.16	.031	1.005	1.113
Va#va	0.99	<.01	-1.94	.052	0.990	1.000
Vfq*	0.91	0.02	-4.89	<.001	0.879	0.946
Vfq#vfq	1.00	<.01	-3.26	.001	0.996	0.999
Full-thickness macular hole (1=Yes; 0=No)	0.23	0.12	-2.87	.004	0.085	0.629
Constant	0.33	0.16	-2.26	.024	0.125	0.864
			Outcome Model Visual Acuity Improvement (VA-I)			
Treatment (1=ocriplasmin; 0=sham)	2.50	1.03	2.21	.027	1.109	5.624
Med, VMA resolution (1=Yes; 0=No)	5.75	2.85	3.53	<.001	2.175	15.186
Age*	0.92	0.02	-3.53	<.001	0.877	0.963
Age#age	0.99	<.01	-1.64	.101	0.995	1.000
Full-thickness macular hole (1=Yes; 0=No)	0.49	0.25	-1.40	.162	0.179	1.332
Female sex (1=Yes; 0=No)	0.44	0.17	-2.08	.037	0.208	0.953
Med#Ftmh Yes#Ftmh present	0.14	0.11	-2.43	.015	0.031	0.690
constant	0.36	0.17	-2.22	.027	0.147	0.899
			Outcome Model Visual Function Response (VF-I)			
Treatment (1=ocriplasmin; 0=sham)	4.09	1.57	3.67	<.001	1.926	8.672
Med, VMA resolution (1=Yes; 0=No)	2.06	0.83	1.81	.071	0.940	4.529
Age*	0.96	0.02	-2.43	.015	0.930	0.992
Epiretinal membrane (1=No; 0=Yes)	2.16	0.97	1.71	.087	0.894	5.228
Full-thickness macular hole (1=Yes; 0=No)	0.15	0.06	-4.86	<.001	0.068	0.320
Female sex (1=Yes; 0=No)	0.53	0.20	-1.71	.087	0.257	1.097
Constant	0.27	0.13	-2.79	.005	0.111	0.681

*Continuous covariates were centered on the mean value of the total study population (age: 69.1; va: 63.1; and vfq: 78.9). The second column shows the regression results expressed in odds ratio, using an indicator coding scheme with 0 as the reference group. An odds ratio (OR) represents the constant effect of a covariate on the odds that the outcome will occur. An OR=1 means the covariate is not associated with the dependent variable after adjustment for the remaining variables. An OR>1 means that an increase in the covariate is associated with an increase in the odds of the dependent variable. An OR<1 means that an increase in the covariate is associated with a decrease in the odds of the dependent variable. SE: Standard errors are based on the robust estimator of variance. Z statistic is the result of a Wald chi-square. P-value provides the 2-tailed p-value used in testing the null hypothesis that the OR=1. The 95% confidence interval (CI) indicates how much uncertainty we have in our estimates. If the CI includes 1 there is evidence that the OR differs from 1.

Table S5.2: Total treatment effect of ocriplasmin on mediator and vision outcomes – with(out) additional covariate adjustments

Model	*Adjustment*	*OR*	*95% CI*	*P value*
**Vitreomacular resolution, VMAR**	1	12.26	4.433 - 33.907	<.001
	2	16.17	5.283 - 49.496	<.001
**Visual function questionnaire improvement, VFQ-I ***	3	1.96	0.765 - 5.017	.161
	4	2.50	0.937 - 6.688	.067
**Visual acuity improvement, VA-I †**	5	2.50	1.109 - 5.624	.027
	6	3.10	1.295 - 7.396	.011
**Visual function response VF-I ‡**	7	4.09	1.926 - 8.672	<.001
	8	4.09	1.863 - 8.980	<.001

Covariate adjustments:
For VMAR: (1) Age, Focal adhesion, Epiretinal membrane (ERM), Treatment by Age interaction; (2) Additional adjustment with Lens, Full-Thickness Macular Hole (FTMH), Sex, Visual Acuity (VA) and Visual Function Questionnaire composite score (VFQ-25cs).
For vision outcome models: *VFQ-I (3): FTMH, VA, VAxVA, VFQ-25cs, VFQ-25csxVFQ-25cs; (4) additional adjustment with Focal adhesion, Lens, ERM and Sex. †VA-I (5): Age, AgexAge, FTMH, Sex, VMAR by FTMH interaction; (6) additional adjustment with Focal adhesion, Lens, ERM, VA and VFQ-25cs. ‡VF-I (7): Age ERM, FTMH, Sex; (8) additional adjustment with Focal, Lens, VA and VFQ-25cs.

Table S5.3: Predicted probability of vision improvement by treatment (t) and VMAR status (incl. 95%CI)

	VFQ-I (%)		*VA-I (%)*		*VF-I (%)*	
	Probability	95% CI	Probability	95% CI	Probability	95% CI
VMAR=0, t=0	14.2	6.2 - 22.3	14.0	6.9 – 21.2	18.0	10.1 – 25.8
VMAR=0, t=1	22.2	14.5 - 30.0	27.3	17.4 – 37.3	42.4	32.0 – 52.8
VMAR=1, t=0	27.8	11.3 – 44.2	33.4	17.2 49.6	29.3	12.9 – 45.6
VMAR=1, t=1	38.5	28.9 – 48.1	49.1	37.6 – 60.6	57.2	45.8 – 68.5

VMAR: vitreomacular adhesion resolution; VMAR=0, t=0: no VMA resolution, sham treatment; VMAR=0, t=1: no VMA resolution, ocriplasmin treatment; VMAR=1, t=0: VMA resolution, sham treatment: VMAR=1, t=1: VMA resolution, ocriplasmin treatment; VFQ-I: visual function questionnaire improvement; VA-I: visual acuity improvement; VF-I: visual function improvement.
