# Supplementary material for: A Causal Inference Approach to Mediation Analysis in Vitreomacular Traction: How Much Does Traction Resolution Mediate Functional Outcomes?
Source: J Mark Access Health Policy. 2024 Sep 30;12(4):280–93. doi: 10.3390/jmahp12040022 (PMC11503358; doi:10.3390/jmahp12040022)
Supplement: Supplementary file 1 [file jmahp-12-00022-s001.zip › S6 Model specifications for mediator and outcome models.pdf]

S6: Model specifications for mediator and outcome models

Mediator and outcome model specifications were built using the general strategy for model selection by Collet, and tested for model adequacy using the Hosmer-Lemeshow goodness-of-fit statistic.

Mediator model

Stata command:
logit Med rt c.age rt#c.age erm focal, nolog vsquish

```
Logistic regression                              Number of obs     =        217
                                                 LR chi2(5)        =      90.85
                                                 Prob > chi2       =     0.0000
Log likelihood = -90.274014                      Pseudo R2         =     0.3347
------------------------------------------------------------------------------
         Med |      Coef.   Std. Err.      z    P>|z|     [95% Conf. Interval]
-------------+----------------------------------------------------------------
          rt |      2.506      0.523     4.79   0.000        1.481       3.532
         age |      0.041      0.050     0.82   0.413       -0.057       0.138
    rt#c.age |
Ocriplasmin  |     -0.170      0.056    -3.03   0.002       -0.280      -0.060
         erm |      2.166      0.648     3.34   0.001        0.895       3.437
       focal |      1.310      0.791     1.66   0.098       -0.241       2.861
       _cons |     -5.793      1.036    -5.59   0.000       -7.824      -3.762
```

Stata command:
estat gof, group(15) all table

Logistic model for Med, goodness-of-fit test

```
  (Table collapsed on quantiles of estimated probabilities)
  +--------------------------------------------------------+
  | Group |   Prob | Obs_1 | Exp_1 | Obs_0 | Exp_0 | Total |
  |-------+--------+-------+-------+-------+-------+-------|
  |     1 | 0.0091 |     0 |   0.1 |    15 |  14.9 |    15 |
  |     2 | 0.0251 |     1 |   0.2 |    13 |  13.8 |    14 |
  |     3 | 0.0608 |     1 |   0.6 |    14 |  14.4 |    15 |
  |     4 | 0.0800 |     0 |   1.3 |    18 |  16.7 |    18 |
  |     5 | 0.0894 |     2 |   1.0 |     9 |  10.0 |    11 |
  |-------+--------+-------+-------+-------+-------+-------|
  |     6 | 0.1240 |     4 |   1.8 |    12 |  14.2 |    16 |
  |     7 | 0.1716 |     2 |   2.1 |    12 |  11.9 |    14 |
  |     8 | 0.2283 |     1 |   2.9 |    13 |  11.1 |    14 |
  |     9 | 0.3316 |     6 |   5.7 |    13 |  13.3 |    19 |
  |    10 | 0.4224 |     2 |   3.5 |     7 |   5.5 |     9 |
  |-------+--------+-------+-------+-------+-------+-------|
  |    11 | 0.5784 |     6 |   7.7 |     9 |   7.3 |    15 |
  |    12 | 0.6729 |    12 |  12.2 |     7 |   6.8 |    19 |
  |    13 | 0.7271 |     9 |   8.5 |     3 |   3.5 |    12 |
  |    14 | 0.7970 |    13 |  12.4 |     3 |   3.6 |    16 |
  |    15 | 0.9488 |    10 |   8.9 |     0 |   1.1 |    10 |
  +--------------------------------------------------------+

       number of observations =       217
             number of groups =        15
```

```
Hosmer-Lemeshow chi2(13) =        13.42
             Prob > chi2 =         0.4162
```

Outcome model VF-I

Stata command:
logit VF-I Med age erm mh rt sex, nolog vsquish

```
Logistic regression                             Number of obs     =        217
                                                LR chi2(6)        =      58.26
                                                Prob > chi2       =     0.0000
Log likelihood = -115.23544                     Pseudo R2         =     0.2018
------------------------------------------------------------------------------
        VF-I |      Coef.   Std. Err.      z    P>|z|     [95% Conf. Interval]
-------------+----------------------------------------------------------------
         Med |      0.729      0.402     1.81   0.070       -0.060       1.517
         age |     -0.045      0.017    -2.62   0.009       -0.079      -0.011
         erm |      0.875      0.421     2.08   0.037        0.051       1.700
          mh |     -1.926      0.393    -4.90   0.000       -2.697      -1.155
          rt |      1.408      0.409     3.44   0.001        0.607       2.209
         sex |     -0.710      0.372    -1.91   0.056       -1.439       0.020
       _cons |     -1.341      0.496    -2.70   0.007       -2.312      -0.369
```

Stata command:
estat gof, group(10) all table

Logistic model for VF-I, goodness-of-fit test

```
  (Table collapsed on quantiles of estimated probabilities)
  +--------------------------------------------------------+
  | Group |   Prob | Obs_1 | Exp_1 | Obs_0 | Exp_0 | Total |
  |-------+--------+-------+-------+-------+-------+-------|
  |     1 | 0.0746 |     0 |   1.1 |    22 |  20.9 |    22 |
  |     2 | 0.1545 |     4 |   2.5 |    18 |  19.5 |    22 |
  |     3 | 0.2172 |     5 |   4.3 |    18 |  18.7 |    23 |
  |     4 | 0.2866 |     4 |   5.1 |    16 |  14.9 |    20 |
  |     5 | 0.3314 |    11 |   6.9 |    11 |  15.1 |    22 |
  |-------+--------+-------+-------+-------+-------+-------|
  |     6 | 0.4245 |     5 |   8.6 |    18 |  14.4 |    23 |
  |     7 | 0.5123 |     9 |   9.5 |    11 |  10.5 |    20 |
  |     8 | 0.6215 |    14 |  13.0 |     9 |  10.0 |    23 |
  |     9 | 0.7671 |    14 |  16.2 |     9 |   6.8 |    23 |
  |    10 | 0.9167 |    17 |  15.8 |     2 |   3.2 |    19 |
  +--------------------------------------------------------+

       number of observations =       217
             number of groups =        10
      Hosmer-Lemeshow chi2(8) =        10.48
                  Prob > chi2 =         0.2332
```

Outcome model VA-I

Stata command:
logit VA-I Med age c.age#c.age mh rt sex Med#mh, nolog vsquish

Logistic regression

	Number of obs	=	217
	LR chi2(7)	=	59.84
	Prob > chi2	=	0.0000
Log likelihood = -99.904232	Pseudo R2	=	0.2305

**VA-I**	Coef.	Std. Err.	z	P>|z|	[95% Conf.	Interval]
Med	1.749	0.502	3.48	0.000	0.765	2.733
age	-0.085	0.022	-3.86	0.000	-0.128	-0.042
c.age#c.age	-0.002	0.001	-1.62	0.105	-0.005	0.000
mh	-2.646	0.621	-4.26	0.000	-3.862	-1.430
rt	0.915	0.440	2.08	0.038	0.052	1.778
sex	-0.810	0.409	-1.98	0.048	-1.611	-0.008
Med#mh						
No#FTMH present	1.930	0.785	2.46	0.014	0.391	3.470
Yes#FTMH absent	0.000	(omitted)				
Yes#FTMH present	0.000	(omitted)				
_cons	-1.017	0.476	-2.14	0.033	-1.949	-0.084

Stata command:
estat gof, group(10) all table

Logistic model for VA-I, goodness-of-fit test
(Table collapsed on quantiles of estimated probabilities)

Group	Prob	Obs_1	Exp_1	Obs_0	Exp_0	Total
1	0.0729	1	1.0	21	21.0	22
2	0.0993	1	1.9	21	20.1	22
3	0.1340	3	2.7	19	19.3	22
4	0.1721	3	3.5	19	18.5	22
5	0.2011	4	3.9	17	17.1	21
6	0.2342	6	4.8	16	17.2	22
7	0.2623	6	5.3	15	15.7	21
8	0.4579	9	8.1	13	13.9	22
9	0.7705	14	15.0	10	9.0	24
10	0.9185	15	15.8	4	3.2	19

number of observations = 217
number of groups = 10
Hosmer-Lemeshow chi2(8) = 1.72
Prob > chi2 = 0.9884

Outcome model VFQ-I

Stata command:
logit VFQ-I Med c.va c.va#c.va vfq c.vfq#c.vfq mh, nolog vsquish

Logistic regression

	Number of obs	=	217
	LR chi2(6)	=	58.90
	Prob > chi2	=	0.0000
Log likelihood = -92.299603	Pseudo R2	=	0.2419

**VFQ-I**	Coef.	Std. Err.	z	P>|z|	[95% Conf.	Interval]
Med	1.291	0.406	3.18	0.001	0.495	2.088
va	0.057	0.028	2.08	0.038	0.003	0.112
c.va#c.va	-0.005	0.003	-1.70	0.089	-0.011	0.001
vfq	-0.097	0.021	-4.53	0.000	-0.138	-0.055
c.vfq#c.vfq	-0.003	0.001	-2.90	0.004	-0.004	-0.001
mh	-1.480	0.497	-2.98	0.003	-2.455	-0.506
_cons	-0.674	0.331	-2.04	0.041	-1.322	-0.026

Stata command:
estat gof, group(10) all table

Logistic model for VFQ-I, goodness-of-fit test

(Table collapsed on quantiles of estimated probabilities)

Group	Prob	Obs_1	Exp_1	Obs_0	Exp_0	Total
1	0.0128	0	0.1	22	21.9	22
2	0.0394	0	0.6	22	21.4	22
3	0.0705	1	1.2	21	20.8	22
4	0.1212	3	2.0	18	19.0	21
5	0.1866	4	3.4	18	18.6	22
6	0.2810	8	5.2	14	16.8	22
7	0.3609	3	6.9	18	14.1	21
8	0.4794	8	9.2	14	12.8	22
9	0.5631	11	11.4	11	10.6	22
10	0.8427	16	14.0	5	7.0	21

number of observations = 217
number of groups = 10
Hosmer-Lemeshow chi2(8) = 7.86
Prob > chi2 = 0.4470
