# Supplementary material for: A Causal Inference Approach to Mediation Analysis in Vitreomacular Traction: How Much Does Traction Resolution Mediate Functional Outcomes?
Source: J Mark Access Health Policy. 2024 Sep 30;12(4):280–93. doi: 10.3390/jmahp12040022 (PMC11503358; doi:10.3390/jmahp12040022)
Supplement: Supplementary file 1 [file jmahp-12-00022-s001.zip › S7 Likelihood Ratio Tests for outcome models with vs without treatment-mediator interaction.pdf]

S7: Likelihood Ratio Tests for outcome models with vs. without treatment-mediator interaction

Logistic regression

Number of obs	= 218
LR chi2(7)	= 56.94
Prob > chi2	= 0.0000
Pseudo R2	= 0.1959

Log likelihood = -116.85078

**VF-I**	Coef.	Std. Err.	z	P>|z|	[95% Conf.	Interval]
Med	0.324	1.247	0.26	0.795	-2.120	2.767
rt	1.355	0.431	3.15	0.002	0.511	2.199
**rt*Med**	0.450	1.315	0.34	0.732	-2.127	3.027
age	-0.039	0.017	-2.33	0.020	-0.073	-0.006
erm	0.768	0.409	1.88	0.061	-0.034	1.570
mh	-1.914	0.392	-4.88	0.000	-2.682	-1.145
sex	-0.646	0.369	-1.75	0.080	-1.368	0.077
_cons	-1.249	0.501	-2.50	0.013	-2.231	-0.268

Logistic regression

Number of obs	= 218
LR chi2(6)	= 56.82
Prob > chi2	= 0.0000
Pseudo R2	= 0.1955

Log likelihood = -116.91174

**VF-I**	Coef.	Std. Err.	z	P>|z|	[95% Conf.	Interval]
Med	0.724	0.401	1.81	0.071	-0.062	1.510
rt	1.408	0.405	3.48	0.001	0.615	2.201
age	-0.040	0.017	-2.39	0.017	-0.073	-0.007
erm	0.771	0.410	1.88	0.060	-0.033	1.575
mh	-1.913	0.391	-4.89	0.000	-2.679	-1.147
sex	-0.633	0.367	-1.73	0.084	-1.352	0.086
_cons	-1.292	0.488	-2.65	0.008	-2.248	-0.335

Akaike's information criterion and Bayesian information criterion

Model	N	ll(null)	ll(model)	df	AIC	BIC
sub	218	-145.3208	-116.9117	7	247.8235	271.5149
full	218	-145.3208	-116.8508	8	249.7016	276.7775

Note: BIC uses N = number of observations. See [R] BIC note.

Likelihood-ratio test LR chi2(1) = 0.12

(Assumption: sub nested in full) **Prob > chi2 = 0.7270**

Logistic regression

Number of obs =	217
LR chi2(8) =	59.87
Prob > chi2 =	0.0000
Log likelihood = -99.887392	
Pseudo R2 =	0.2306

**VA-I**	Coef.	Std. Err.	z	P>|z|	[95% Conf. Interval]	
Med	1.516	1.380	1.10	0.272	-1.190	4.221
rt	0.886	0.467	1.90	0.058	-0.029	1.802
**rt*Med**	0.260	1.430	0.18	0.856	-2.542	3.062
age	-0.084	0.022	-3.82	0.000	-0.127	-0.041
age*age	-0.002	0.001	-1.61	0.107	-0.005	0.000
mh	-0.715	0.511	-1.40	0.161	-1.716	0.285
sex	-0.816	0.411	-1.99	0.047	-1.621	-0.012
Med*mh	-1.932	0.786	-2.46	0.014	-3.473	-0.391
_cons	-0.996	0.489	-2.04	0.042	-1.953	-0.038

Logistic regression

Number of obs =	217
LR chi2(7) =	59.84
Prob > chi2 =	0.0000
Log likelihood = -99.904232	
Pseudo R2 =	0.2305

**VA-I**	Coef.	Std. Err.	z	P>|z|	[95% Conf. Interval]	
Med	1.749	0.502	3.48	0.000	0.765	2.733
rt	0.915	0.440	2.08	0.038	0.052	1.778
age	-0.085	0.022	-3.86	0.000	-0.128	-0.042
age*age	-0.002	0.001	-1.62	0.105	-0.005	0.000
mh	-0.716	0.511	-1.40	0.161	-1.717	0.285
sex	-0.810	0.409	-1.98	0.048	-1.611	-0.008
Med*mh	-1.930	0.785	-2.46	0.014	-3.470	-0.391
_cons	-1.017	0.476	-2.14	0.033	-1.949	-0.084

Akaike's information criterion and Bayesian information criterion

Model	N	ll(null)	ll(model)	df	AIC	BIC
sub	217	-129.8245	-99.90423	8	215.8085	242.8476
full	217	-129.8245	-99.88739	9	217.7748	248.1939

Note: BIC uses N = number of observations. See [R] BIC note.

Likelihood-ratio test LR chi2(1) = 0.03

(Assumption: sub nested in full) **Prob > chi2 = 0.8544**

Logistic regression

Number of obs	=	217
LR chi2(9)	=	61.35
Prob > chi2	=	0.0000
Pseudo R2	=	0.2520

Log likelihood = -91.074725

**VFQ-I**	Coef.	Std. Err.	z	P>|z|	[95% Conf.	Interval]
Med	1.545	1.351	1.14	0.253	-1.102	4.192
rt	0.786	0.517	1.52	0.128	-0.227	1.799
**rt*Med**	-0.600	1.414	-0.42	0.672	-3.371	2.172
age	-0.008	0.019	-0.43	0.665	-0.046	0.029
va	0.053	0.029	1.82	0.068	-0.004	0.109
va*va	-0.005	0.003	-1.65	0.099	-0.011	0.001
vfq	-0.091	0.021	-4.29	0.000	-0.133	-0.050
vfq*vfq	-0.002	0.001	-2.67	0.008	-0.004	-0.001
mh	-1.509	0.504	-2.99	0.003	-2.497	-0.521
_cons	-1.147	0.478	-2.40	0.016	-2.085	-0.210

Logistic regression

Number of obs	=	217
LR chi2(8)	=	61.18
Prob > chi2	=	0.0000
Pseudo R2	=	0.2513

Log likelihood = -91.160771

**VFQ-I**	Coef.	Std. Err.	z	P>|z|	[95% Conf.	Interval]
Med	1.002	0.459	2.18	0.029	0.103	1.901
rt	0.708	0.480	1.47	0.140	-0.233	1.650
age	-0.007	0.019	-0.38	0.704	-0.044	0.030
va	0.054	0.029	1.87	0.062	-0.003	0.110
va*va	-0.005	0.003	-1.63	0.102	-0.011	0.001
vfq	-0.092	0.021	-4.30	0.000	-0.134	-0.050
vfq*vfq	-0.002	0.001	-2.70	0.007	-0.004	-0.001
mh	-1.487	0.501	-2.97	0.003	-2.470	-0.505
_cons	-1.102	0.462	-2.39	0.017	-2.008	-0.197

Akaike's information criterion and Bayesian information criterion

Model	N	ll(null)	ll(model)	df	AIC	BIC
sub	217	-121.7513	-91.16077	9	200.3215	230.7406
full	217	-121.7513	-91.07473	10	202.1495	235.9484

Note: BIC uses N = number of observations. See [R] BIC note.

Likelihood-ratio test LR chi2(1) = 0.17

(Assumption: sub nested in full) **Prob > chi2 = 0.6783**
