# Supplementary material for: A Causal Inference Approach to Mediation Analysis in Vitreomacular Traction: How Much Does Traction Resolution Mediate Functional Outcomes?
Source: J Mark Access Health Policy. 2024 Sep 30;12(4):280–93. doi: 10.3390/jmahp12040022 (PMC11503358; doi:10.3390/jmahp12040022)
Supplement: Supplementary file 1 [file jmahp-12-00022-s001.zip › S8 Mediation effect estimates in the presence of treatment mediator interaction.pdf]

S8: Mediation effect estimates in the presence of treatment-mediator interaction

The outcome models that included a treatment by mediator (TxM) interaction did not result in a significant improvement in model fit. The main text of the paper therefore reports the mediation results based on the assumption of no-interaction. Nevertheless, to better understand the impact of TxM interaction on mediation, we include the results in Table E.

Causal effect estimates for the 3 vision outcomes

Table E displays the causal effect estimates. The mediation effect represents the effect of ocriplasmin on vision outcomes through ocriplasmin's treatment effect on VMAR. In OASIS, the *mediated effect under treatment condition IE(1)* represents the average treatment effect on the vision outcome that is transmitted by the change in VMAR induced by ocriplasmin. The IE(1) answers the counterfactual question: What change would occur to the probability of the vision outcome if the level of VMAR changed from what would be realized under sham *M*(0), to the level of VMAR that would be observed under ocriplasmin *M*(1), while holding the treatment constant at ocriplasmin (*t=1*). More specifically, the mediated effect is a comparison of the probability of a vision improvement when the level of VMAR were those that what would occur with ocriplasmin vs the probability of a vision improvement when the level of VMAR were those that would occur with sham but in fact occurred during receipt of ocriplasmin therapy. The *direct effect under control condition DE(0)* compares the effect of ocriplasmin (*t=1*) vs. sham (*t=0*) on vision outcomes while holding the level of VMAR constant at the level that would be realized under sham treatment *M*(0). The total treatment effect is broken down into the *mediated effect IE(1)* and *direct effect DE(0).* Total treatment effect represents how much (%) the vision outcomes would change overall, for a change in the exposure from sham to ocriplasmin.

Table A: Causal effect estimates for the binary vision outcomes – impact treatment by mediator interaction

Average effects (%)	No T by M interaction	With T by M interaction
	VFQ-I*	
**IE (1)† [95% CI]**	5.7‡ [1.16 , 10.86]	7.8 [-4.29 , 19.22]
**DE (0)† [95% CI]**	8.3 [-3.30 , 19.53]	8.1 [-3.90 , 18.57]
**TTE† [95% CI]**	13.9‡ [2.61, 24.23]	15.9 [-3.39, 31.58]
**% TTE mediated via IE (1)**	40.0 [22.75 , 167.28]	44.3 [-215.90 , 328.88]
	VA-I*	
**IE (1)† [95% CI]**	11.8‡ [4.99 , 19.41]	9.8 [-1.94 , 20.81]
**DE (0)† [95% CI]**	12.2‡ [1.53 , 22.95]	11.5 [-0.32 , 22.45]
**TTE† [95% CI]**	23.9‡ [12.15 , 34.97]	21.3‡ [2.28 , 36.78]
**% TTE mediated via IE (1)**	48.9 [33.67 , 96.77]	43.8 [25.29 , 218.17]
	VF-I*	
**IE (1)† [95% CI]**	5.2 [-0.31 , 11.31]	2.4 [-10.00 , 15.74]
**DE (0)† [95% CI]**	24.1‡ [11.58 , 36.56]	22.9‡ [9.52 , 35.56]
**TTE† [95% CI]**	29.3‡ [17.81 , 40.20]	25.4‡ [4.46 , 42.21]
**% TTE mediated via IE (1)**	17.7 [12.98 , 29.28]	9.25 [5.64 , 44.03]

*Risk difference at month 24
† IE (1): indirect effect under treatment condition, ocriplasmin (*t*=1); DE (0): direct effect under control condition, sham (*t*=0); TTE: total treatment effect (difference in observed % of ocriplasmin-treated participants with a vision improvement vs. sham-treated participants.
‡ Results with significant values at p<0.05.

The dynamics of mediation can be appreciated when we compare the scenario with vs. without T by M interaction. When interaction is introduced, the mediated effect under ocriplasmin condition

changes from a significant risk difference (RD) of 5.7% to a nonsignificant RD of 7.8% for VFQ-I, and from 11.8% to a nonsignificant RD of 9.8%. When adding a T by M interaction term, the causal effects for VF-I remain nonsignificant.
